# "Is this sedation?” – a Group Delphi process to define cut-off doses and dosing intervals for potentially sedating drugs in palliative care

**DOI:** 10.1186/s12904-025-01781-8

**Published:** 2025-06-07

**Authors:** Sabine H. Krauss, Constanze Rémi, Claudia Bausewein, Jeremias Bazata, Alina Grebe, Christoph Ostgathe, Jan Schildmann, Eva Schildmann, the iSedPall study group

**Affiliations:** 1 Department of Palliative Medicine, LMU University Hospital, LMU Munich, Marchioninistr. 15, Munich, 81377 Germany; 2 Department of Palliative Medicine, CCC Erlangen – EMN, University Hospital Erlangen, Friedrich-Alexander-Universität Erlangen-Nürnberg (FAU), Krankenhausstraße 12, Erlangen, 91054 Germany; 3 Institute for History and Ethics of Medicine, Interdisciplinary Center for Health Sciences, Martin Luther University Halle-Wittenberg, Magdeburger Straße 8, Halle (Saale), 06112 Germany; 4 Palliative Medicine, Faculty of Medicine, University of Augsburg, Stenglinstraße 2, Augsburg, 86156 Germany

**Keywords:** Palliative sedation, Deep sedation, Light sedation, Palliative care, Suffering, Administration and dosage, Sedatives, Delphi study, Group Delphi

## Abstract

**Background:**

In palliative care, it can be challenging to distinguish between reduced consciousness related to the illness and sedation due to a potentially sedating drug (intended, or unintended). These differentiations are important because unintended sedation requires consideration of alternative treatment options, and intentional sedation demands compliance with guidelines. The aim of the study, which was part of the consortium project iSedPall, was to determine cut-off values for drugs’ doses/dosing intervals which are expected to result in defined depth of sedation/continuous effect.

**Methods:**

Group Delphi conducted in Germany with prior online survey. Based on a review of the literature, statements regarding cut-off values for drugs´ doses/dosing intervals which are expected to result in a defined depth of sedation/continuous effect were developed for 11 drugs. Consensus was defined as ≥ 75% agreement. Statements with lower agreement entered the next round of discussion. Between the rounds (5 small groups, 3 – 4 participants each), the results were presented and discussed. If necessary, statements were adapted for the following round. Participating experts were physicians, pharmacists, and nurses experienced in palliative care, mostly with over 10 years of professional experience.

**Results:**

25/30 invited experts participated in the online survey, 17 in the Group Delphi. 12/33 statements were consented in the survey. The initial questionnaire for the Group Delphi comprised 22 statements on ten drugs. After three rounds, consensus was reached for all statements, determining cut-off doses/dosing intervals for lorazepam, midazolam, diazepam, levomepromazine, haloperidol, melperone, pipamperone, propofol, dexmedetomidine, and trazodone.

**Conclusions:**

This study for the first time provides evidence- and expert consensus-based data to support clinical judgements regarding sedating effects of a range of potentially sedating drugs commonly used in palliative care.

**Supplementary Information:**

The online version contains supplementary material available at 10.1186/s12904-025-01781-8.

## Introduction/background

In palliative care, reduced consciousness is often (partly) caused by the illness. Besides, many of the drugs used in palliative care can have sedating effects [1]. Frequently, there is a seamless transition from the use of potentially sedating drugs without the reduction of consciousness, e.g. for anxiolysis, towards light or even deep sedation [2, 3]. In these cases, sedation may be an unwanted effect or one that is (potentially not explicitly) intended – often without a clear ‘starting point’ of the latter. Consequently, there is uncertainty when (intentional) sedation begins [4, 5]. Intentional sedation to relieve suffering/so-called ‘palliative sedation’ is an important but still debated treatment option for unbearable suffering from one or more refractory symptoms [6–9]. A current terminology proposes the term “intentional sedation”, which is defined as “result or process of sedating a patient as a means of achieving a previously defined treatment goal” [10].

While there is increasing agreement on which substances and dosages should be used for intentional sedation to relieve suffering, little attention has been paid to situations in which sedation occurs unintentionally [2, 11, 12]. When sedation is unintentionally induced, e.g. by gradually increasing doses of drugs with sedative effects, then patients, relatives and professionals are faced with a fait accompli. This can lead to ambiguities within the team, a lack of communication with patients and relatives, and non-compliance with relevant guidelines in general [4, 13, 14]. For example, prior information of patients and relatives about sedation, discussion of worries, queries and wishes for the time of sedation as well as seeking informed consent is only possible before intentional sedation is started [15, 16]. Other best practice components emphasised by relevant guidelines [12, 17], such as meeting the prerequisites for indication and decision-making, as well as adequate monitoring of sedation and documentation of each step of the procedure, also depend on actually discerning a starting point or at least acknowledging that sedation has already been started unintentionally. Conversely, in case of unintentional sedation that is considered as an unwanted adverse effect, it is good practice to consider possible alternatives to reverse the sedative effect.

Therefore, it is important to support professionals in their judgement regarding sedative effects at certain doses and dosing intervals – as an essential prerequisite for consecutive decisions. However, the evidence base for this is very limited. The scarce available data are mainly drug dosages recommended for intentional sedation to relieve suffering in palliative care, and these are mainly based on retrospective studies of heterogeneous patient populations or on expert opinions [2, 18, 19]. Therefore, the aim of this study was to determine and consent cut-off values (‘red flags’) for doses and dosing intervals which are expected to result in a defined depth of sedation (light or deep sedation) or continuous effect, respectively, for selected potentially sedating drugs commonly used in palliative care.

## Methods

### Study design

This study was part of the consortium project iSedPall, funded by the German Ministry of Research and Education (BMBF 01GY2020 A-C) [20]. iSedPall aimed to develop and pilot a multi-modal intervention that operationalises previously developed recommendations for the use of sedative drugs in specialist palliative care into practical tools for healthcare professionals in inpatient and home care settings [21, 22]. The Group Delphi reported here was an important component of the project and served as foundation for the development of a clinical decision support tool (reported elsewhere).

**Fig. 1:**
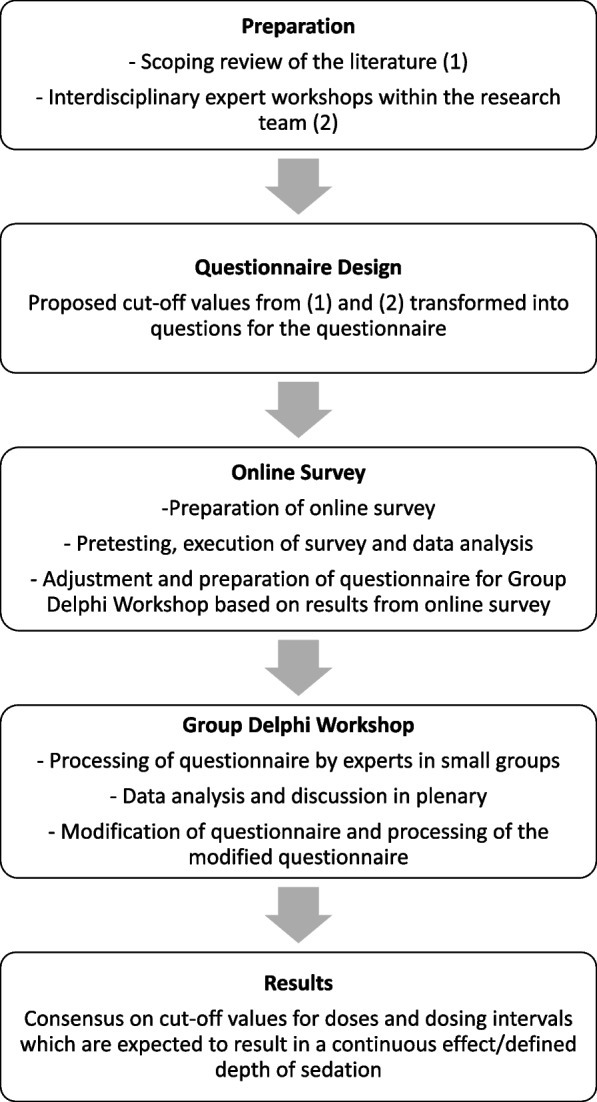
Stages of the Group Delphi process

We conducted a Group Delphi study with a preparatory online survey in Germany. The online survey was aimed to identify topics which could be consented this way and topics of dissent. The latter then entered the Group Delphi process (see Fig. 1). The study was performed and is reported according to the recommendations for Conducting and Reporting of Delphi Studies (also see Additional file 1 – CREDES checklist) [23].

For this topic with little existing evidence, the Group Delphi study design has the following advantages: A Group Delphi involves real-time, face-to-face discussions among participants, allowing for immediate clarification of ideas and fostering deeper understanding. This contrasts with the Classical Delphi, which relies on asynchronous communication through questionnaires [24–27]. The interactive nature of a Group Delphi accelerates the consensus process, as participants can directly address disagreements, refine their views and modify the Group Delphi statements accordingly during the session [25, 27, 28]. This was particularly valuable, as the task – to establish cut-off dose values and dosing intervals for potentially sedating drugs which are expected to result in a defined depth of sedation – involved complex deliberations and inferences from recommendations aimed for other purposes (e.g. drug doses recommended for ‘sedation’, often without explicitly specifying the depth of sedation, or drug doses for ‘easing agitation’). In general, the iterative process in a Delphi serves as a form of calibration, refining group responses to achieve a more reliable consensus [24, 28].

### Selection of experts

The experts were invited based on a predefined sample frame consisting of two main criteria: professional group and setting (see Table 1).

**Table 1:** Sample frame

	**Online Survey**	**Workshop**
Number of participants	25 (± 5)	18 (± 2)
Professional group		
Physician	20 (± 4)	14 (± 2)
Nurse	2 (± 1)	2 (± 1)
Pharmacist	3 (± 1)	2 (± 1)
Setting		
Inpatient	15 (± 3)	10 (± 2)
Community	10 (± 1)	8 (± 1)
Clinical Background		
Palliative Care	20 (± 4)	14 (± 2)
Intensive care	5 (± 1)	4 (± 1)
International working experience	5 (± 1)	4 (± 1)
Professional experience in the field		
> 10 years	20 (± 4)	14 (± 2)
< 10 years	5 (± 1)	4 (± 1)

The sample was based on the rationale that experts should have comprehensive knowledge and experience with potentially sedating drugs. In addition, the sample was aimed to gather a broad variety of opinions. A high proportion of physicians was chosen as they are the professional group that carries the responsibility for drug prescribing in Germany and are prone to have experience regarding the level of sedation in relation to doses. Experts from inpatient settings were thought to have more experience with a broader variety of potentially sedating drugs than experts from community settings. It was thought appropriate to involve experts from intensive care units to obtain valuable opinions and insights into their experience on the effect of sedative drugs, including those which (so far) are not regularly used in palliative care. Experts with international work experience were included because they might have a broader view on the topic and therefore be able to bring in different perspectives. As the Group Delphi was planned to be held in German, experts from all over Germany and German-speaking countries were considered suitable. 30 experts were invited to participate in the online survey. The same experts were invited to the Group Delphi with the target to have 16–20 experts in the workshop, as recommended [24, 28]. The experts were invited due to their role as stakeholders or their exceptional experience in palliative care or drug safety. Two members of the research team (ES project lead and physician, CR pharmacist) identified potential members of the purposive sample. Based on their own status as experts in the field with long-term expansive experience and a large professional network, they assessed the status of each potential member of the sample regarding his or her status as stakeholder or exceptional expert.

### Preparatory online survey

The substances considered were potentially sedating drugs commonly used in palliative care, independent of being recommended for intentional sedation to relieve suffering in palliative care or not. The primary therapeutic use of these substances in clinical practice (e.g. anxiolysis) was not relevant to the objective of this project. Drugs had to meet the following criteria to be included: 1a) frequently used with the intention of sedation or 1b) frequently used for treatment of agitation in palliative care. In addition to one of these criteria, a further condition had to be fulfilled: 2a) at least limited data available that allow conclusions to be drawn about the doses at which sedative effects can be expected or 2b) sufficient clinical experience in the team to assess doses with sedating effects.

Relevant potentially sedating drugs and content for the statements were identified based on a scoping review of the literature and the medical and pharmaceutical expertise within the research team. The scoping review included a search in PubMed and Pubpharm. Search strings mainly consisted of two topic blocks with MeSH terms and keywords:"sedation"(e.g. palliative sedation, procedural sedation, analgosedation) and"drugs"and/or keywords (e.g. midazolam, lorazepam and/or dosage, cut-off, threshold). Boolean operators were used to combine the terms (also see Additional file 3 – Search terms for literature review). A filter was used to eliminate results before the year 2000. In addition, we searched for international guidelines and recommendations on sedation. For all drugs, the manufacturers'information was also searched for dose information regarding any kind of potential sedating effects, e.g. sedation, procedural sedation, sleep, anxiolysis, etc.

11 drugs met the inclusion criteria. Based on the information from the available literature and their own extensive clinical experience, a pharmacist and a physician of the research team developed the statements for the survey questionnaire. Differences in opinion were resolved by discussion with a third experienced physician of the team. For the questionnaire, the following definitions were used: The depth of sedation was defined according to the RASS-PAL scale [29], in line with recommendations for intentional sedation which had been developed for the project that preceded the iSedPall project: light sedation referring to −1 and −2 on the RASS-PAL scale and deep sedation referring to −3 to −5 on the RASS-PAL scale [17]. The RASS-PAL scale is based on the Richmond Agitation-Sedation Scale used in intensive care. It is well established in Palliative Care [2, 12]. To ensure that all participants have a similar understanding of the patients referred to – and therefore to ensure that similar clinical judgements were possible, a ‘standard patient’ was used. This ‘standard patient’ was defined as a patient in a palliative care situation, i.e. with life-threatening illness, female or male, approximately 60 years old, body weight approximately 70 kg, no substance abuse (especially benzodiazepines) currently or in the past, no impaired consciousness prior to the administration of the potentially sedating drug, no impairment of the blood–brain barrier, no severe renal or hepatic impairment (GFR ≤ 30 ml/min or ChildPugh B or C).

The questionnaire had a clearly arranged design. Following a brief information on the study, type and content of the questions and the type of patients referred to (the defined ‘standard patient’) were explained and a German translation of the RASS-PAL-scale [29] provided. The survey pages for all 11 drugs were structured in the same way: three pre-formulated statements, each followed by a short explanatory statement why this statement was chosen, a field for free text comments, and listed references. For each drug, the first statement provided the time interval for a sustained clinical effect (e.g.: “For lorazepam, because of the pharmacokinetic characteristics of the substance, a sustained clinical effect (not necessarily sedation) is to be expected for the above mentioned ‘standard patient’ when it is administered every 8–12 h.”). The second statement provided details on dosages for which a light level of sedation is to be expected (−1/−2 on RASS-PAL scale, e.g.:”For the administration of 2 mg lorazepam (independent from route of application, bioavailability > 90% [with relevant reference] as single dose, sedation of at least −1 on the RASS-PAL scale (light sedation) is to be expected in > 50% of the above mentioned ‘standard patient’.”). The third statement indicated doses for a sedation level of −3 (RASS-PAL) and deeper (“deep sedation”) to be expected. After each statement, participants were asked to rate their degree of consent for the statement on a 4-point Likert scale (agree completely, rather agree, rather do not agree, and do not agree at all). After thorough discussion, we used a 4-point Likert scale as opposed to a 5- or 7-point scale. The rationale was that we wanted the respondents to decide between the tendency to agree or disagree by not offering a middle category, and that the granularity of four options was enough for our study aim. Additionally, participants were asked to rate the degree of confidence regarding their response, also on a 4-point Likert scale (completely confident, rather confident, rather not confident, and not confident at all). In a field for free text comments, respondents could give a more detailed answer. This option was provided to gain further insight into the experts’ knowledge and their experience with potentially sedating drugs, e.g., alternative dosages or a statement how much (or little) experience they had with a drug. At the end of the questionnaire, there were also two open-ended questions. The first asked whether there were additional drugs in the participants´ opinion, for which they saw a relevant risk for a seamless transition to sedation when applying this substance, and for which, therefore, such cut-off values should also be determined. The second question asked whether they wanted to make any other comment, e.g. a topic associated with this survey that should be discussed in the Group Delphi workshop. At the end of the questionnaire, nine demographic questions were asked (also see Additional file 2 – Questionnaire of the online survey).

The online questionnaire was pretested in 3 rounds with a total of 10 people who were not part of the Group Delphi expert group: A first round with the pharmacist and physicians of the iSedPall research alliance, a second with scientific staff of the iSedPall research alliance, and a final round with professionals from the local palliative care unit. On the basis of the pretests, minor adjustments were made to the wording and layout of the survey. The option to go back in the online questionnaire was also implemented, based on the feedback from the pretests.

The survey was conducted from March to April 2022 using Limesurvey [30].

**Fig. 2:**
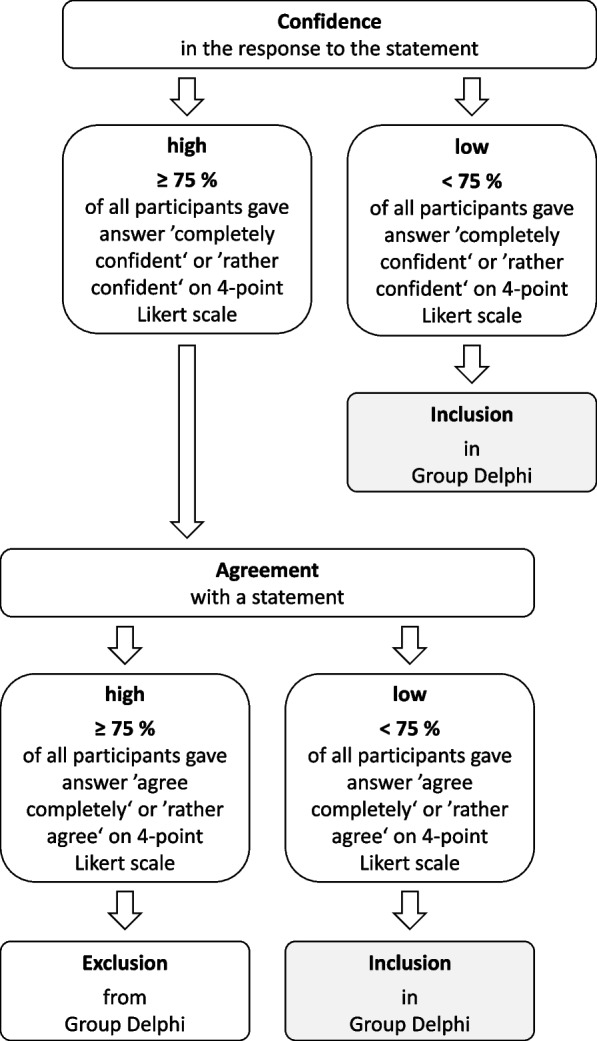
Algorithm for selection of questions for the Group Delphi workshop

A pre-defined algorithm was used to decide whether the statement was considered to have gained consent or whether it should be part of the Group Delphi workshop (see Fig. 2). To analyse the free text comments and the open-ended questions, content analysis was used [31, 32]. The questionnaire for the first round of the Group Delphi was based on the results of the survey.

### Group Delphi workshop

The workshop was conducted in June 2022 as a 1.5-day event supported by an independent professional facilitator. The participants for the groups (5 groups with 3–4 participants in each group) were selected in advance and then recomposed for each round. Every time, we aimed for a good mix of professional backgrounds in each group and for a new ‘mix’ of participants for every group compared to the previous rounds. The questionnaire followed the same structure as the online survey. The groups were asked to mark their agreement with each statement on a 4-point Likert scale. In contrast to the online survey, the option"other"was removed, as well as the questions regarding confidence: it was intended to prompt a decision; at the same time, the users were invited to comment on their decision or to state any other opinion in the section “comments” directly below, e.g. give their proposal for an alternative statement. The workshop started with an explanation about the aim, research method, and the questions. The groups were then separated, each receiving one questionnaire. Ideally, the result of the discussion was group consensus. Disagreements, inconsistencies or comments could be listed. The answers from each group were collected by the researchers and entered into prepared tables (Microsoft Excel). Afterwards, the results were presented and discussed in the plenary. The plenary discussions were led by a professional facilitator to ensure a balanced discussion. In the event of disagreement, joint proposals were developed, e.g. for rewording statements, which were then discussed again in the small groups. This approach also minimised the risk of groupthink. Based on the results and the plenary discussion, the questions were modified in the plenum if necessary and taken to the next group meeting. Statements with approval or another form of consent within the plenum were removed from the questionnaire. This procedure was repeated until all statements were consented.

Ethical approval has been obtained from the Local Research Ethics Committee of the Medical Faculty at Friedrich-Alexander-Universität Erlangen-Nürnberg (No. 21–381-B from 24 th of November 2021) and from the Local Research Ethics Committee of the Medical Faculty at Ludwig-Maximilians-University Munich (No. 22-0026 from 18 th of February 2022).

## Results

### Preparatory online survey

Thirty experts were invited to participate in the preparatory online survey, 25 questionnaires were completed. For a description of the sample see Table 2.

**Table 2:** Sample description

	Online Survey		Workshop	
Number of participants	25		17	
Women	16		10	
Professional group^a^	*Setting*		*Setting*	
	*Inpatient*	*Community*	*Inpatient*	*Community*
Physician	17	9	11	9
Nurse	2	2	1	1
Pharmacist	2	0	3	2
Clinical Background				
Palliative medicine	21		16	
Intensive care	4		1	
International working experience	7		4	
Professional experience in the field				
> 10 years	19		12	
< 10 years	6		5	

^a^Multiple responses were permitted

Based on the definition shown above (Fig. 2) 12/33 statements received an agreement in the online survey. 21 statements received either a low percentage of agreement or participants were not confident enough in their response to the statement. Lorazepam was the only drug for which all statements received approval in the online survey. All other drugs had at least one statement with a high percentage of disagreement or low confidence. Low confidence regarding the response to a statement was the commonest reason for a statement not to be approved. Phenobarbital received the lowest rate of consent (< 30%).

In the free text comments, participants for example stated no or little experience with a drug or made comments regarding the dosage or effect of a drug. Within the corresponding open question section, several additional drugs were mentioned, including opioids, promethazine, quetiapine, risperidone, mirtazapine, and amitriptyline. General remarks included questions regarding the ‘standard patient’ or drug interaction. All comments provided in the open question sections were carefully reviewed and discussed by the research team. It was decided that no additional drugs should be included in the Group Delphi process as these were isolated entries without suggestions of cut-off dosages or underlying evidence. As other comments also represented isolated views, the questionnaire was not changed, except for one statement about propofol, where an additional dose range was given. This resulted in one more statement which was added to the questionnaire for the Group Delphi, leading to a total of 22 statements to be rated in the Group Delphi.

### Group Delphi workshop

The subsequent Group Delphi workshop was conducted with 17 participants over a total of three rounds. After round 1, ten of the 22 statements (the 21 from the questionnaire of the online survey and the additional one for Propofol, based on the results from the online survey) could be consented and the questionnaire for round 2 included 12 statements; in round 3, four statements were discussed again.

After the three Delphi rounds, agreement was finally reached on all 22 statements, including agreement on three rejections (Table 3). In combination with the results of the online survey, this resulted in a total of 35 statements on 11 drugs. The summarised results were sent to all participants after the workshop.

**Table 3:**
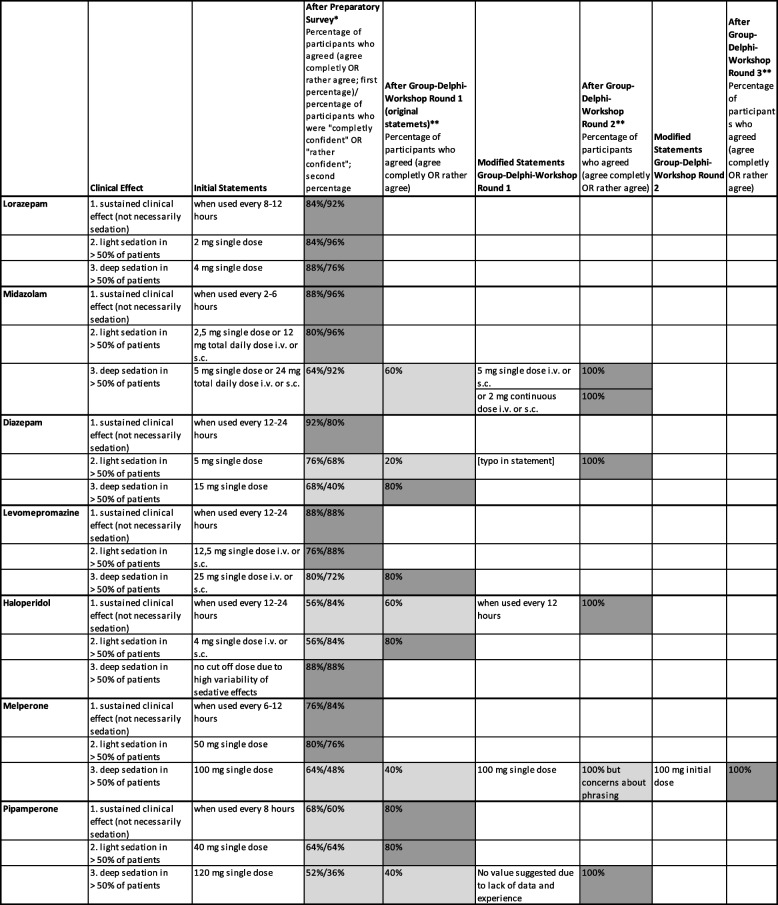

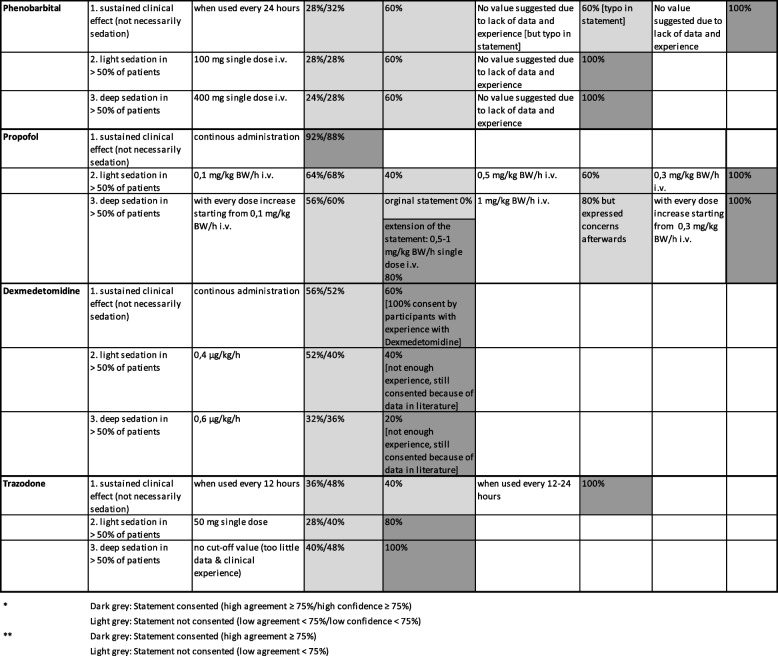
Statements and results of the preparatory online survey and the three Group Delphi rounds

### Main results for different drugs

#### Midazolam

Some participants judged that a total daily dose for midazolam should be provided rather than an infusion rate of a continuous parenteral administration. In the plenary, it was decided to split the original statement into two statements (midazolam parenteral single dose, midazolam continuous parenteral administration) and to discuss them in the following round.

#### Haloperidol

In the first round, no consensus could be reached regarding the dosing interval, so the statement was rediscussed in the plenary and second round. In the plenary, it was agreed to change the dosing interval to "every 12 h"(instead of "every 12–24 h"). The statement was adapted accordingly.

#### Melperone

In the plenary discussion of the first round, it was discussed whether a cut-off dose would be useful, as melperone does not normally lead to deep sedation. No consensus could be reached regarding the sedative effects of different doses. Therefore, it was agreed to replace the word "single dose" with the term "initial dose (i.e. without prior dosage titration)". This was to address participants' concerns that 100 mg as a single dose after prior titration without strong sedative effects is not uncommon in practice.

#### Pipamperone

In the plenary discussion, many participants emphasised the lack of clinical experience with higher doses. The plenary therefore decided to rephrase the statement so that no cut-off value would be set due to insufficient data and clinical experience.

#### Phenobarbital

After the first round, the plenary decided not to provide any drug dosages because it is hardly used in clinical practice and because of very limited evidence. This decision was discussed again in the next round and finally consented.

#### Propofol

Based on the first round and plenary discussion, the dosages potentially leading to light sedation were increased and eventually consented. In contrast to the original statement of the online survey (no cut-off value for deep sedation), a cut-off value statement for deep sedation was agreed upon after intensive discussion in the plenary and in small groups. According to this, any dose increase above the cut-off value for light sedation may have the potential to cause deep sedation.

#### Dexmedetomidine

After intensive discussion about the current and future relevance of dexmedetomidine as well as the (relatively) short history of use, and despite the lack of consensus in the Group Delphi, it was decided not to discuss the statements further, but to keep the statements and treat them as consented based on available evidence and clinical recommendations (but not experience).

#### Trazodone

Based on the discussion, the dosing interval for a sustained clinical effect was changed to 24 h after the first round; this statement was then taken into the next round and was consented. The statements on cut-off doses were consented after the first round.

With regard to the four drugs with low agreement and low confidence concerning all three statements in the online survey (dexmedetomidine, phenobarbital, pipamperone and trazodone), the decisions were influenced by the following factors: Dexmedetomidine is specifically approved for sedation (in intensive care). The doses correspond to the manufacturer's instructions for specific levels of sedation. The group therefore consented the inclusion of dexmedetomidine, despite the lack of personal experience. For phenobarbital, there was no information from the manufacturer, no data on concrete dosages in the literature regarding sedation, and no personal experience. For pipamperone and trazodone, the manufacturer's information and personal experience of the expert group suggested that at least light sedation can be expected with the doses included in the questionnaire. However, there was no information about possible deep sedation.

## Discussion

### Main results

This study for the first time provides evidence- and expert consensus-based data to support clinical judgements regarding sedating effects of a range of potentially sedating drugs commonly used in palliative care. Research and recommendations to date have focused on intentional sedation to relieve suffering. Yet the highly relevant area of the transition between therapies such as anxiolysis without sedative effect, and sedation as an unintended or ‘accepted’, but not explicitly acknowledged as ‘intended’, concomitant effect has not been examined in detail [3]. Many drugs used in palliative care have a central depressant effect. As part of symptom management, sedation is likely to occur [1]. At the same time, reduction of consciousness is also commonly caused by other, mostly illness-related, factors in this seriously ill patient population. As the transition from not sedated to sedated is fluid and highly dependent on individual patient factors, we believe that it is necessary to provide support for clinical judgements and respective decisions regarding the current and future care of the patient. Therefore, the aim of our project was to provide warnings as to when sedation is to be expected although other therapeutic intentions are pursued. In this particular context, our findings should be regarded as information to support clinical judgement and decision-making in an individual situation, rather than as a guideline.

We intentionally included drugs that are not recommended for sedation but are commonly used in palliative care and may have relevant sedating properties at least above a certain dose range, e.g. haloperidol. A large number of other medications regularly used in palliative care may have sedating effects, and, if given concomitantly to drugs included in this study, may, of course, contribute to the overall sedating effect. This must certainly be taken into account in clinical practice. Our aim, however, was to develop specific cut-off values, above which a certain depth of sedation (or continuous effect, respectively) can be expected. For this reason, potentially sedating drugs for which it was not possible to consent dose thresholds – due to insufficient evidence and experience of the experts in the Group Delphi or due to large inter-individual variability of the relation between dose and effect – were not included, e.g. opioids and other benzodiazepines apart from lorazepam and midazolam, or had to be removed from the list of drugs with consented statement, such as phenobarbital. Our results provide guidance for the anticipation of possible sedating effects or for the detection of already established unintended sedation and can help to avoid unintentionally induced sedation.

The dose recommendations for sedation in palliative care in respective international guidelines were, together with other sources from the literature, also taken into consideration for the determination of cut-off doses for potential sedating effects. This was, naturally, only possible for those drugs which are recommended for the purpose of intentional sedation in palliative care. In these cases, the cut-off dose values determined in our study (see Table 3) are comparable to the respective recommendations [12]. However, it must be emphasised – once again – that the aim of this project was to raise awareness for potentially unwanted or unintended sedation, and not to provide dose recommendations for intentional sedation.

### Standard patient

For the compilation and discussion of doses and dosing intervals, we decided to predefine a ‘standard patient’, being aware that such a patient does not exist. The patient population in palliative care is heterogeneous – related to age, gender, a variety of underlying diseases, co-morbidities, co-medications, etc. The potential influence of these factors on drug dosages must be determined and discussed in the individual context. However, the standard patient can represent the respective basis for the discussion. Future research might explore the relation between dose and sedating effect for different patient subgroups. This could enable a more detailed and nuanced understanding of variations of sedating effects at given doses depending on individual patient characteristics such as gender, age, body weight, organ function, and interactions with other drugs. Based on these findings, specific cut-off values and, based on that, also specific dose recommendations for drugs recommended for intentional sedation, could be determined for different patient subgroups [9].

### Chosen study design and strengths and limitations of this study

Theoretically, the ideal study design to meet our aim would have been a ‘cut-off dose-finding’ study for each individual drug to empirically determine the respective cut-off doses for light or deep sedation and dosing intervals for continuous effect – comparable to phase 2 studies for new drugs. However, this is ethically and practically not possible in a palliative care population. The second-best option would have been observational prospective studies. However, especially for rarely applied drugs, it would take a long time to recruit a sufficient number of patients, and standardisation of assessments, e.g. for level of sedation, and, importantly, regarding doses in relation to the individual characteristics of the patients, is very challenging. Retrospective analyses of routine data from case records would not have met the aim, as routine data lack routinely documented, structured assessments of consciousness/sedation levels, let alone assessments according to RASS-PAL, as well as structured documentation of patient characteristics relevant to the effect of a given dose for this individual patient. Therefore, the choice of a Group Delphi procedure, based on a literature review, was chosen as the best option to achieve robust evidence- and expert-based results in a reasonable time frame.

In a Delphi process – either classical or Group Delphi – the quality of results relies on the selection of experts, which can lead to a bias if the group lacks diversity or expertise in relevant areas [24]. Therefore, a diverse sample with highly experienced experts was chosen and achieved. A possible limitation is the inclusion of experts only from two German-speaking countries. However, by carefully selecting experts from different settings, centres, professions, and disciplines, and including experts with international expertise, we believe that the best possible consensus has been achieved.

In comparison to a classical Delphi, the disclosure of judgements in the Group Delphi can be a limitation when participants do not share their opinion and join the majority view [24, 28]. Therefore, a competent moderation is essential. We tried to mitigate this risk by employing a professional facilitator and strived to create a friendly atmosphere.

The selected Group Delphi format has been demonstrated to be successful in achieving consensus in circumstances where scientific evidence on a given topic is limited [33]. It provided an excellent opportunity to collect expert-based information in addition to the results of the literature review, in the form of ‘live’ discussed clinical experience and expert opinions as well as knowledge of additional literature regarding relevant drugs and dosages.

The cut-off values thus determined can serve as a basis for future prospective empirical studies to create more robust evidence.

## Conclusion

In this project, we determined and consented cut-off values and dosing intervals for selected drugs with sedating effects above which a defined depth of sedation and continuous effect are to be expected. The cut-off doses and dosing intervals can support the judgement whether a patient´s reduced consciousness is probably (partly) a consequence of the drug treatment, and therefore promote considerations for alternative treatment options with less sedating effect. They can also serve as ‘red flags’ to raise awareness when sedation has to be expected with drugs used in other indications, e.g. anxiolysis. Consequently, they can facilitate the compliance with guidelines for intentional sedation in palliative care, such as providing adequate information about sedation to the patient and relatives, exploring wishes for the time of sedation and gaining informed consent. Third, the obtained cut-off values of doses for light sedation to be expected and the dosing intervals for continuous effect may inform dose recommendations for intentional sedation to relieve suffering.

In a next step, the data are used for the development of a so-called ‘warning list’ to support clinical judgements regarding the possible sedating and continuous effects of a drug (reported elsewhere) and of dose recommendations for the start of intentional sedation to relieve suffering in palliative care (also reported elsewhere). The ‘warning list’ and the dose recommendations are part of a multimodal intervention developed and piloted by the consortium project iSedPall. Importantly, the cut-off values cannot replace an individual clinical assessment. They are intended to support a more conscious use of potentially sedating drugs in palliative care, a correct and explicit labelling of the respective treatment and, in consequence, the compliance with relevant guidelines and general principles of best practice.

## Supplementary Information

Supplementary Material 1.

Supplementary Material 2.

Supplementary Material 3.

## Data Availability

All data generated or analysed during this study are included in this published article.
