# Correction: “Is this sedation?” – a Group Delphi process to define cut-off doses and dosing intervals for potentially sedating drugs in palliative care

**DOI:** 10.1186/s12904-025-01865-5

**Published:** 2025-08-19

**Authors:** Sabine H. Krauss, Constanze Rémi, Claudia Bausewein, Jeremias Bazata, Alina Grebe, Christoph Ostgathe, Jan Schildmann, Eva Schildmann, on behalf of the iSedPall study group

**Affiliations:** 1 Department of Palliative Medicine, LMU University Hospital, LMU Munich, Marchioninistr. 15, Munich, 81377 Germany; 2 Department of Palliative Medicine, CCC Erlangen– EMN, University Hospital Erlangen, Friedrich-Alexander-Universität Erlangen-Nürnberg (FAU), Krankenhausstraße 12, Erlangen, 91054 Germany; 3 Institute for History and Ethics of Medicine, Interdisciplinary Center for Health Sciences, Martin Luther University Halle-Wittenberg, Magdeburger Straße 8, Halle (Saale), 06112 Germany; 4 Palliative Medicine, Faculty of Medicine, University of Augsburg, Stenglinstraße 2, Augsburg, 86156 Germany

**Correction: BMC Palliat Care 24**,** 159 (2025)**

**https://doi.org/10.1186/s12904-025-01781-8**

Following publication of the original article [1], the author reported that the following sentence found on both page 5 and page 11 should be updated:

From

"Ethical approval has been obtained from the Local Research Ethics Committee of the Medical Faculty at Ludwig-Maximilians-University Munich (No. 21–381-B from 24 th of November 2021)."

 to 

"Ethical approval has been obtained from the Local Research Ethics Committee of the Medical Faculty at Friedrich-Alexander-Universität Erlangen-Nürnberg (No. 21–381-B from 24 th of November 2021) and from the Local Research Ethics Committee of the Medical Faculty at Ludwig-Maximilians-University Munich (No. 22-0026 from 18 th of February 2022)."

The original article has been updated.
