# Supplementary material for: "Is this sedation?” – a Group Delphi process to define cut-off doses and dosing intervals for potentially sedating drugs in palliative care
Source: BMC Palliat Care. 2025 Jun 7;24:159. doi: 10.1186/s12904-025-01781-8 (PMC12144799; doi:10.1186/s12904-025-01781-8)

Dokumentation LimeSurvey – Vorabbefragung

Ansicht der Nutzer:innen

Inhaltsverzeichnis

1. Beschreibung und Willkommenstext 2
2. Hinweise 3
3. Fragen zu Medikamenten 5
   - 3.1 Lorazepam 5
   - 3.2 Midazolam 9
   - 3.3 Diazepam 14
   - 3.4 Levomepromazin 18
   - 3.5 Haloperidol 23
   - 3.6 Melperon 27
   - 3.7 Pipamperon 31
   - 3.8 Phenobarbital 35
   - 3.9 Propofol 39
   - 3.10 Dexmedetomidin 43
   - 3.11 Trazodon 47
4. Weitere sedierende Medikamente 51
5. Zusätzliche Aspekte für das Gruppen-Delphi 51
6. Demographische Daten 52
7. Abschlusstext 56
8. Allgemeines zum Layout 57
   - 8.1 Prozentleiste 57
   - 8.2 Navigationshilfe: Fragenindex 57

1. **Beschreibung und Willkommenstext**

Online-Befragung „Sedierende Medikamente / Gezielte Sedierung"

iSedPall - Entwicklung und Machbarkeitsprüfung einer multimodalen Intervention für den Gebrauch sedierender Medikamente in der spezialisierten Palliativversorgung

Sehr geehrte Damen und Herren,

als Expertin bzw. Experte für sedierende Medikamente haben Sie eine Einladung zur Teilnahme an dieser Vorabbefragung als Teil eines Gruppendelphi-Verfahrens erhalten. Herzlichen Dank, dass Sie dem Link gefolgt sind.

Diese Online-Befragung ist Teil des vom BMBF geförderten Verbundprojekts „iSedPall – Entwicklung und Machbarkeitsprüfung einer multimodalen Intervention für den Gebrauch sedierender Medikamente in der spezialisierten Palliativversorgung (SPV)". Sie dient der Vorbereitung auf einen Expert*innen-Workshop und soll ermöglichen, Bereiche zu identifizieren, bei denen bereits großer Konsens bzw. noch großer Dissens oder Diskussionsbedarf besteht. Ziel der Online-Befragung und des Expert*innen-Workshops ist die Diskussion und Konsentierung von Medikamentendosierungen zu verschiedenen Kategorien des Gebrauchs sedierender Medikamente und gezielter Sedierung zur Leidenslinderung in der Palliativversorgung.
Die nun folgende Befragung enthält jeweils 3 Aussagen zu 11 potenziell sedierenden Medikamenten und Begründungen dieser Statements.

Sie werden etwa 30 – max. 60 min für die Befragung benötigen. Sie können innerhalb der Befragung jederzeit vor oder zurück gehen. Sie können die Bewertung der Statements auch unterbrechen und später fortsetzen. Zum Schluss der Befragung werden wir Sie um einige für unsere Auswertung relevante demographische Daten bitten.

Diese Online-Befragung ist bis **einschließlich 18.04.2022** aktiv geschaltet. Die Beantwortung der Fragen erfolgt anonym. *LimeSurvey* speichert keine identifizierbaren Daten der Teilnehmenden. Mit Ihrer Teilnahme erklären Sie sich einverstanden, dass Ihre Angaben in anonymisierter Form verwendet werden.

Bei Fragen zu dieser Online-Befragung wenden Sie sich bitte an:

Jerri Bazata: jeremias.bazata@med.uni-muenchen.de
Sabine Krauss: sabine.krauss@med.uni-muenchen.de

Mit herzlichen Grüßen

Eva Schildmann, Constanze Rémi, Jerri Bazata, Sabine Krauss und Claudia Bausewein

2. Hinweise

Hinweise

Im Folgenden bitten wir um Ihre Bewertung von jeweils **drei Aussagen pro potentiell sedierendem Medikament**, bezogen auf die folgenden **zwei Leitfragen**:

1. Leitfrage: Wann ist mit einem anhaltenden klinischen Effekt (nicht notwendigerweise Sedierung) des Medikaments zu rechnen (außer bei kontinuierlicher parenteraler Applikation)? (Aussage 1)

2. Leitfrage: „Bei welcher Dosis/welchem Dosisschema ist bei mehr als 50% der behandelten Patient*innen mit leichter (Aussage 2) bzw. tiefer (Aussage 3) Sedierung zu rechnen?“

Für die Definition von "leichter" und "tiefer" Sedierung nutzen wir die **RASS-PAL** Skala mit den Werten -1 bzw. -3 (s. unten).

Alle Vorschläge in den Aussagen beziehen sich auf folgende **„Standard-Patient*in“**:

Patient*in in Palliativversorgung, also mit lebensbegrenzender Erkrankung, weiblich oder männlich, ca. 60 Jahre alt, Körpergewicht (KG) ca. 70 kg, kein Substanzabusus (v.a. Benzodiazepine) in der Vorgeschichte oder aktuell, keine Bewusstseinseinschränkung vor Gabe des potentiell sedierenden Medikaments, keine Einschränkung der Blut-Hirn-Schranke, keine stärkere Einschränkung der Nieren- oder Leberfunktion (GFR = 30ml/min oder ChildPugh B oder C).

RASS-PAL

Die RASS-PAL-Skala (Bush et al. 2014) ist eine Skala zur Bestimmung der Bewusstseinslage/Sedierungstiefe.

RASS-PAL	Beschreibung	Sedierungsgrad lt. SedPall-Empfehlungen
1 – 5	**s. RASS-PAL**	Keine Sedierung
0	**Aufmerksam und ruhig**	
-1<br>(s. Aussage 2)	**Schläfrig:**<br>Nicht ganz aufmerksam, bleibt aber auf Ansprache wach (Augen öffnen/Blickkontakt: mind. 10 Sekunden)	Leichte Sedierung
-2	**Leichte Sedierung:**<br>Erwacht kurz mit Blickkontakt auf Ansprache hin (weniger als 10 Sekunden)	
-3<br>(s. Aussage 3)	**Mäßige Sedierung:**<br>Irgendeine Bewegung (Augen oder Körper) oder Augenöffnung auf Ansprache (aber kein Blickkontakt)	Tiefe Sedierung
-4	**Tiefe Sedierung:**<br>Keine Reaktion auf Ansprache, aber irgendeine Bewegung (Augen oder Körper) oder Augenöffnung auf leichte Berührung hin	
-5	**Nicht erweckbar:**<br>Keine Reaktion auf Ansprache oder auf leichte Berührung hin	

Wir schlagen im Folgenden Cut-off-Werte für die potentiell sedierenden Medikamente vor, die
1a) häufig zumindest auch mit Intention von Sedierung verwendet werden
oder
1b) die häufiger bei Unruhe in der Palliativmedizin eingesetzt werden

UND

2a) Für die es zumindest rudimentäre Daten gibt, die Rückschlüsse darauf erlauben, bei welchen Dosierungen mit sedierenden Effekten zu rechnen ist
oder
2b) es ausreichend klinische Erfahrung zur Beurteilung von Dosierungen mit sedierenden Effekten gibt.

Bitte beachten Sie: die aufgeführten Applikationswege und Indikationen der einzelnen Medikamente weichen teilweise von den für dieses Medikament zugelassenen ab (Off-Label-Use).

Literatur

Bush, S. H., Grassau, P. A., Yarmo, M. N., Zhang, T., Zinkie, S. J., & Pereira, J. L. (2014). *The Richmond Agitation-Sedation Scale modified for palliative care inpatients (RASS-PAL): a pilot study exploring validity and feasibility in clinical practice.* BMC palliative care, 13(1), 17.

Forschungsverbund SedPall (Hrsg.) (2021): *Handlungsempfehlung Einsatz sedierender Medikamente in der Spezialisierten Palliativversorgung.* Online verfügbar hier.

3. Fragen zu Medikamenten

3.1 Lorazepam

Lorazepam

Aussage 1: Bei Lorazepam ist aufgrund der pharmakokinetischen Charakteristika der Substanz bei o.g. Standardpatient*innen ein anhaltender klinischer Effekt (nicht notwendigerweise Sedierung) zu erwarten, wenn es **alle 8 - 12 Stunden** angewendet wird.

Plasma-Halbwertszeit: 12 – 16 Std. [1]

Begründung für Vorschlag des Dosierintervalls für anhaltenden klinischen Effekt: Laut Fachinfo bei Angst-, Spannungs- und Erregungszuständen und damit assoziierten Schlafstörungen 2 – 3 mal tgl. Gabe empfohlen. Wir gehen bei dieser Indikation von einem anhaltenden klinischen Effekt aus, der mit dem angegebenen Dosierintervall erzielt wird.

Bitte wählen Sie die zutreffende Antwort für jeden Punkt aus:

Aussage 1 Lorazepam						Wie sicher fühlen Sie sich in der Beantwortung der Frage?			
stimme vollkommen zu	stimme eher zu	stimme eher nicht zu	stimme überhaupt nicht zu	Sonstiges (bitte Freitext am Ende der Seite nutzen)		vollkommen sicher	eher sicher	eher nicht sicher	überhaupt nicht sicher
○	○	○	○	○		○	○	○	○

ANSICHT DER NUTZER:INNEN

<u>Aussage 2</u>: Bei der Gabe von **2 mg** Lorazepam (Applikationsweg-unabhängig, Bioverfügbarkeit >90% [1]) als Einzeldosis ist bei > 50% der o.g. „Standardpatient*innen" mit einer Sedierung von mind. -1 auf der RASS-PAL-Skala (leichte Sedierung) zu rechnen.

<u>Grundlagen für diesen Vorschlag:</u> Klinische Erfahrung und verfügbare Literatur (s. unten)

Bitte wählen Sie die zutreffende Antwort für jeden Punkt aus:

**Aussage 2 Lorazepam**						**Wie sicher fühlen Sie sich in der Beantwortung der Frage?**			
**stimme vollkommen zu**	**stimme eher zu**	**stimme eher nicht zu**	**stimme überhaupt nicht zu**	**Sonstiges (bitte Freitext am Ende der Seite nutzen)**		**vollkommen sicher**	**eher sicher**	**eher nicht sicher**	**überhaupt nicht sicher**
○	○	○	○	○		○	○	○	○

Aussage 3: Bei der Gabe von **4 mg** Lorazepam (Applikationsweg-unabhängig, Bioverfügbarkeit >90% [1]) als Einzeldosis ist bei > 50% der o.g. „Standardpatient*innen" mit einer Sedierung von mind. -3 auf der RASS-PAL-Skala (mäßige Sedierung) zu rechnen.

Grundlagen für diesen Vorschlag: Klinische Erfahrung und verfügbare Literatur (s. unten)

Bitte wählen Sie die zutreffende Antwort für jeden Punkt aus:

Aussage 3 Lorazepam						Wie sicher fühlen Sie sich in der Beantwortung der Frage?			
stimme vollkommen zu	stimme eher zu	stimme eher nicht zu	stimme überhaupt nicht zu	Sonstiges (bitte Freitext am Ende der Seite nutzen)		vollkommen sicher	eher sicher	eher nicht sicher	überhaupt nicht sicher
○	○	○	○	○		○	○	○	○

Freitextfeld für Ihre Kommentare:

Literatur:

[1]: Rémi, C., Bausewein, C., Charlesworth, S., Wilcock, A., & Howard, P. (Eds.). (2022). *Arzneimitteltherapie in der Palliativmedizin*. Elsevier Health Sciences.

[2]: Fachinformation Tavor Tabletten, Pfizer (Stand 05/2021):

- zur "Sedierung vor diagnostischen sowie vor und nach operativen Eingriffen":
  - 2 bis 4mg ca. 1 bis 2 Stunden vor Eingriff;
  - Bei älteren oder geschwächten Pat. Reduktion der Tagesgesamt-Dosis um ca. 50%

[3]: European Association for Palliative Care (EAPC) recommended guideline for the use of sedation in palliative care (DRAFT):

- Für „palliative sedation“ (ohne Angabe der Sedierungstiefe)
  - als Initial-Bolus für „palliative sedation“ 1 – 3 mg s.c. oder i.v.
  - Erhaltungsdosis: entweder Bolus 1 – 3 mg s.c./i.v. alle 2-4h oder 1 - 5mg/h s.c. oder i.v. cont.

[4]: Quill, T. E., & Byock, I. R. (2000). Responding to intractable terminal suffering: the role of terminal sedation and voluntary refusal of food and fluids. ACP-ASIM End-of-Life Care Consensus Panel. American College of Physicians-American Society of Internal Medicine. *Annals of internal medicine*, *132*(5), 408–414.

- "Terminal sedation is the use of high doses of sedatives to relieve extremes of physical distress"
- Übliche Startdosis (aus einigen Referenzen adaptiert): 1- 4mg alle 4 - 6 Stunden oral/buccal oder 0.5- 1mg/h i.v.
- Erhaltungsdosis 4 - 40mg/24h

3.2 Midazolam

Midazolam

Aussage 1: Bei Midazolam ist aufgrund der pharmakokinetischen Charakteristika der Substanz bei o.g. Standardpatient*innen ein anhaltender klinischer Effekt (nicht notwendigerweise Sedierung) zu erwarten, wenn es **alle 2 – 6 Stunden** angewendet wird.

Plasma-Halbwertszeit: 1.5 – 2,5 Stunden bei Gesunden, bei > 60 Jahren bis zu 4-fach verlängert, bei Schwerstkranken bis 6-fach verlängert [1].

Begründung für Vorschlag des Dosierintervalls für anhaltenden klinischen Effekt: Plasma-Halbwerts-zeit [1] und klinische Erfahrung.

Bitte wählen Sie die zutreffende Antwort für jeden Punkt aus:

Aussage 1 Midazolam						Wie sicher fühlen Sie sich in der Beantwortung der Frage?			
stimme vollkommen zu	stimme eher zu	stimme eher nicht zu	stimme überhaupt nicht zu	Sonstiges (bitte Freitext am Ende der Seite nutzen)		vollkommen sicher	eher sicher	eher nicht sicher	überhaupt nicht sicher
○	○	○	○	○		○	○	○	○

<u>Aussage 2</u>: Bei der Gabe von **2,5 mg** Midazolam als Einzeldosis bzw. **12 mg** als Tagesgesamtdosis, jeweils parenteral (i.v. oder s.c.), ist bei > 50% der o.g. „Standardpatient*innen" mit einer Sedierung von mind. -1 auf der RASS-PAL-Skala (leichte Sedierung) zu rechnen.

<u>Grundlagen für diesen Vorschlag:</u> Klinische Erfahrung und verfügbare Literatur (s. unten)

Bitte wählen Sie die zutreffende Antwort für jeden Punkt aus:

Aussage 2 Midazolam						Wie sicher fühlen Sie sich in der Beantwortung der Frage?			
stimme vollkommen zu	stimme eher zu	stimme eher nicht zu	stimme überhaupt nicht zu	Sonstiges (bitte Freitext am Ende der Seite nutzen)		vollkommen sicher	eher sicher	eher nicht sicher	überhaupt nicht sicher
○	○	○	○	○		○	○	○	○

Aussage 3: Bei der Gabe von **5 mg** Midazolam als Einzeldosis bzw. **24 mg** als Tagesgesamtdosis, jeweils parenteral (i.v. oder s.c.), ist bei > 50% der o.g. „Standardpatient*innen" mit einer Sedierung von mind. -3 auf der RASS-PAL-Skala (mäßige Sedierung) zu rechnen.

Grundlagen für diesen Vorschlag: Klinische Erfahrung und verfügbare Literatur (s. unten)

Bitte wählen Sie die zutreffende Antwort für jeden Punkt aus:

Aussage 3 Midazolam						Wie sicher fühlen Sie sich in der Beantwortung der Frage?			
stimme vollkommen zu	stimme eher zu	stimme eher nicht zu	stimme überhaupt nicht zu	Sonstiges (bitte Freitext am Ende der Seite nutzen)		vollkommen sicher	eher sicher	eher nicht sicher	überhaupt nicht sicher
○	○	○	○	○		○	○	○	○

Freitextfeld für Ihre Kommentare:

Literatur:

[1]: Fachinfo Midazolam B. Braun 1 mg/ml Injektions-/Infusionslösung oder Rektallösung, Stand 06/2018.

[2]: Cherny, N. I., Radbruch, L., & Board of the European Association for Palliative Care (2009). European Association for Palliative Care (EAPC) recommended framework for the use of sedation in palliative care. *Palliative medicine*, *23*(7), 581–593.

- Startdosis 0.5 - 1mg/h, 1 - 5mg bei Bedarf
- Übliche Wirkdosis: 1 - 20mg/h

[3]: European Association for Palliative Care (EAPC) recommended guideline for the use of sedation in palliative care (DRAFT):

- Leichte palliative Sedierung (RASS-PAL -1 - -3):
  - Initialdosis: 2.5mg s.c. oder 1.25mg iv
  - Erhaltungsdosis: Auftitrieren von 1mg/h (bei Alter > 60 kg 0,5 mg/h) bis maximal 10mg/h
- Tiefe palliative Sedierung (RASS-Pal -4/-5) (cave: wir suchen den Dosis-cut-off-Wert für RASS-PAL -3!)
  - Initialdosis: 5-10mg s.c. / 2.5-5mg iv
  - Erhaltungsdosis: wie leichte palliative Sedierung

[4]: Imai, K., Morita, T., Yokomichi, N., Kawaguchi, T., Kohara, H., Yamaguchi, T., Kikuchi, A., Odagiri, T., Watanabe, Y. S., Kamura, R., Maeda, I., Kawashima, N., Ito, S., Baba, M., Matsuda, Y., Oya, K., Kaneishi, K., Hiratsuka, Y., Naito, A. S., & Mori, M. (2021). Efficacy of Proportional Sedation and Deep Sedation Defined by Sedation Protocols: A Multicenter, Prospective, Observational Comparative Study. *Journal of pain and symptom management*, *62*(6), 1165–1174.

- Proportional sedation (Ziel: IPOS <=1 & RASS <=0)
  - Startdosis: 0.5 - 2mg/h mit Bolus 0.5 - 2mg
  - Wenn Symptome nicht ausreichend gelindert, dann Dosiserhöhung 10-70% plus Bolus 0.5-2mg alle 2 Stunden
  - Median nach 4 bzw. 24 Std: 1mg/h bzw.1.2mg/h
- Deep sedation (RASS <=-4) (cave: wir suchen den Dosis-cut-off-Wert für RASS-PAL -3!)
  - Startdosis 5 - 10mg/h mit Bolus 0.5 - 2mg
  - Wenn benötigt, Dosiserhöhung um 10-70% plus Bolus 0.5-2mg alle 15-30 min
  - Wenn erwünschte Sedierungstiefe erreicht wurde 1-3mg/h mit Möglichkeit erneuter Steigerung
  - Median "Loading Phase" (bis Erreichen der Sed.Tiefe) 6.4 mg/h
  - Median nach 4 bzw. 24Std: 1.5mg/h bzw.3.1mg /h

[5]: Quill, T. E., & Byock, I. R. (2000). Responding to intractable terminal suffering: the role of terminal sedation and voluntary refusal of food and fluids. ACP-ASIM End-of-Life Care Consensus Panel. American College of Physicians-American Society of Internal Medicine. *Annals of internal medicine*, *132*(5), 408–414.

- "Terminal sedation is the use of high doses of sedatives to relieve extremes of physical distress
- Übliche Startdosis (aus einigen Referenzen adaptiert): 0.5-1.5mg/h, zusätzlich zu Beginn 0.5 mg Bolus
- Erhaltungsdosis 30-100mg/24h

[6]: NCCN (2016): NCCN Clinical Practice Guidelines in Oncology (NCCN Guidelines). Palliative Care (Präsentation).

- Für „Palliative sedation“ bei „unmittelbar sterbenden“ Patient*innen, Sedierungstiefe nicht näher definiert:
  - Initial infusion rate 0.4-0.8 mg/h, range: 20-102 mg/h

[7]: Gamblin, V., Berry, V., Tresch-Bruneel, E., Reich, M., Da Silva, A., Villet, S., Penel, N., & Prod'Homme, C. (2020). Midazolam sedation in palliative medicine: retrospective study in a French center for cancer control. *BMC palliative care*, *19*(1), 85.

- „Proven sedation“/“Bewiesene Sedierung“ (= in Krankenakte "sedation" hinterlegt)
  - Startdosis (Bolus): Median 3.0 mg (range: 0.5-10 mg/h)
  - Erhaltungsdosis: Median 1.0 mg/h (range: 0.2-7 mg/h)
  - Zum Todeszeitpunkt: Median 2 mg/h (range: 0.2-24 mg/h)
- „Probable sedation“/“Wahrscheinliche Sedierung (= Midazolam verabreicht (>= 0.5 mg/h) als Reaktion auf therapierefraktäres Symptom, aber "sedation" nicht dokumentiert)
  - Startdosis (Bolus): 3.5 mg
  - Erhaltungsdosis: Median 1.0 mg/h (range: 0.2-2 mg/h)
  - Zum Todeszeitpunkt: Median 3.0 mg/h (range: 2.5-20 mg/h)

[8]: Sykes, N., & Thorns, A. (2003). Sedative use in the last week of life and the implications for end-of-life decision making. *Archives of internal medicine*, *163*(3), 341–344.

- Retrospective Aktenanalyse, bei der cut-off-Werte für potentiell sedierende Medikamente bestimmt wurden, oberhalb derer “signifikante Sedierung” “wahrscheinliche Folge” war
- Für Midazolam: Tagesgesamtdosis 10mg/24h

3.3 Diazepam

Diazepam

Aussage 1: Bei Diazepam ist aufgrund der pharmakokinetischen Charakteristika der Substanz bei o.g. Standardpatient*innen ein anhaltender klinischer Effekt (nicht notwendigerweise Sedierung) zu erwarten, wenn es **alle 12 - 24** Stunden angewendet wird. (Insbesondere bei Gabe über mehrere Tage ist die einmal tägliche Gabe ausreichend für einen anhaltenden klinischen Effekt.)

Plasma-Halbwertszeit: 1 Std., aktive Metaboliten bis 100 Std [1, 2]

Begründung für Vorschlag des Dosierintervalls für anhaltenden klinischen Effekt: Laut Fachinfo bei Spannungs-, Erregungs- und Angstzuständen 1 – 2 mal tägliche Gabe empfohlen. Wir gehen bei dieser Indikation von einem anhaltenden klinischen Effekt aus, der mit dem angegebenen Dosierintervall erzielt wird.

Laut Fachinfo wird bei regelmäßiger täglicher Einnahme ein Steady state für Diazepam und den wirksamen Metaboliten N-Desmethyldiazepam altersabhängig innerhalb von 5 – 15 Tagen erreicht [2].

Plasma-Halbwertszeit: 1 Std., aktive Metaboliten bis 100 Std [1, 2]

Begründung für Vorschlag des Dosierintervalls für anhaltenden klinischen Effekt: Laut Fachinfo bei Spannungs-, Erregungs- und Angstzuständen 1 – 2 mal tägliche Gabe empfohlen. Wir gehen bei dieser Indikation von einem anhaltenden klinischen Effekt aus, der mit dem angegebenen Dosierintervall erzielt wird.

Laut Fachinfo wird bei regelmäßiger täglicher Einnahme ein Steady state für Diazepam und den wirksamen Metaboliten N-Desmethyldiazepam altersabhängig innerhalb von 5 – 15 Tagen erreicht [2].

Bitte wählen Sie die zutreffende Antwort für jeden Punkt aus:

Aussage 1 Diazepam						Wie sicher fühlen Sie sich in der Beantwortung der Frage?			
stimme vollkommen zu	stimme eher zu	stimme eher nicht zu	stimme überhaupt nicht zu	Sonstiges (bitte Freitext am Ende der Seite nutzen)		vollkommen sicher	eher sicher	eher nicht sicher	überhaupt nicht sicher
○	○	○	○	○		○	○	○	○

Aussage 2: Bei der Gabe von **5 mg** Diazepam (Applikationsweg-unabhängig, Bioverfügbarkeit >90% [3]) als Einzeldosis ist bei > 50% der o.g. „Standardpatient*innen" mit einer Sedierung von mind. -1 auf der RASS-PAL-Skala (leichte Sedierung) zu rechnen.

Grundlagen für diesen Vorschlag: Klinische Erfahrung und verfügbare Literatur (s. unten)

Bitte wählen Sie die zutreffende Antwort für jeden Punkt aus:

Aussage 2 Diazepam						Wie sicher fühlen Sie sich in der Beantwortung der Frage?			
stimme vollkommen zu	stimme eher zu	stimme eher nicht zu	stimme überhaupt nicht zu	Sonstiges (bitte Freitext am Ende der Seite nutzen)		vollkommen sicher	eher sicher	eher nicht sicher	überhaupt nicht sicher
○	○	○	○	○		○	○	○	○

Aussage 3: Bei der Gabe von **15 mg** Diazepam (Applikationsweg-unabhängig, Bioverfügbarkeit >90% [3]) als Einzeldosis ist bei > 50% der o.g. „Standardpatient*innen" mit einer Sedierung von mind. -3 auf der RASS-PAL-Skala (mäßige Sedierung) zu rechnen.

Grundlagen für diesen Vorschlag: Klinische Erfahrung und verfügbare Literatur (s. unten; u.a. unter Berücksichtigung der Dosis für Narkoseeinleitung lt. Fachinfo [2]: 14 -24 mg bei 70 kg KG)

Bitte wählen Sie die zutreffende Antwort für jeden Punkt aus:

Aussage 3 Diazepam						Wie sicher fühlen Sie sich in der Beantwortung der Frage?			
stimme vollkommen zu	stimme eher zu	stimme eher nicht zu	stimme überhaupt nicht zu	Sonstiges (bitte Freitext am Ende der Seite nutzen)		vollkommen sicher	eher sicher	eher nicht sicher	überhaupt nicht sicher
○	○	○	○	○		○	○	○	○

Freitextfeld für Ihre Kommentare:

ANSICHT DER NUTZER:INNEN

Literatur:

[1]: Fachinfo Diazepam Desitin rectal tube (Stand 09/2018).

- Behandlung akuter Angst- Spannungs- und Erregungszustände: rektal 5-10mg Bolus alle 3-4 Stunden bis max. 4 Dosen am Tag
- Prämedikation vor chirurgischen Eingriffen: Am Vorabend 10-20mg rektal, 1 Stunde vor Narkosebeginn 5-10mg

[2]: Fachinfo Diazepam-ratiopharm® Injektionslösung (Stand 10/2018, Version 2).

- Die Narkoseeinleitung erfolgt bei Erwachsenen mit 0,2 – 0,35 mg Diazepam/kg Körpergewicht i. v. – bei Risikopatienten fraktioniert (entspricht bei Körpergewicht 70 kg 14 – 24 mg)

[3]: Rémi, C., Bausewein, C., Charlesworth, S., Wilcock, A., & Howard, P. (Eds.). (2022). *Arzneimitteltherapie in der Palliativmedizin*. Elsevier Health Sciences.

[4]: KNMG (2009). Guideline for Palliative Sedation. Royal Dutch Medical Association (KNMG).

- Palliative sedation (ohne Angabe der Sedierungstiefe, aber vermutlich eher für tiefere Sedierung):
  - 10mg/h rektal bis gewünschte Sedierungstiefe erreicht.
  - Normalerweise 40-60mg/24h

3.4 Levomepromazin

Levomepromazin

Aussage 1: Bei Levomepromazin ist aufgrund der pharmakokinetischen Charakteristika der Substanz bei o.g. Standardpatient*innen ein anhaltender klinischer Effekt (nicht notwendigerweise Sedierung) zu erwarten, wenn es **alle 12 – 24** Stunden angewendet wird.

Plasma-Halbwertszeit: 15 – 30 Std. [1]

Begründung für Vorschlag des Dosierintervalls für anhaltenden klinischen Effekt: Plasma-Halbwertszeit [1] und angegebene Wirkdauer von 12-24 Stunden [1].

Bitte wählen Sie die zutreffende Antwort für jeden Punkt aus:

Aussage 1 Levomepromazin						Wie sicher fühlen Sie sich in der Beantwortung der Frage?			
stimme vollkommen zu	stimme eher zu	stimme eher nicht zu	stimme überhaupt nicht zu	Sonstiges (bitte Freitext am Ende der Seite nutzen)		vollkommen sicher	eher sicher	eher nicht sicher	überhaupt nicht sicher
○	○	○	○	○		○	○	○	○

Aussage 2: Bei der Gabe von **12,5 mg** Levomepromazin s.c. oder i.v. als Einzeldosis ist bei > 50% der o.g. „Standardpatient*innen" mit einer Sedierung von mind. -1 auf der RASS-PAL-Skala (leichte Sedierung) zu rechnen. (Keine Daten zur oralen Gabe verfügbar. Aufgrund der geringen oralen Bioverfügbarkeit (20-50%) [1] ist von jeweils der doppelten Dosis für orale Gabe auszugehen.)

Grundlagen für diesen Vorschlag: Klinische Erfahrung und verfügbare Literatur (s. unten; u.a.: häufig 12,5 mg als Startdosis angegeben, dann aber in 6-h Abstand; letzteres wahrscheinlich für tiefere Sedierung als RASS-PAL-1, bei 6.25 oder 12.5mg/24h noch keine nennenswerten sedierenden Effekte beobachtet [7]).

Bitte wählen Sie die zutreffende Antwort für jeden Punkt aus:

Aussage 2 Levomepromazin						Wie sicher fühlen Sie sich in der Beantwortung der Frage?			
stimme vollkommen zu	stimme eher zu	stimme eher nicht zu	stimme überhaupt nicht zu	Sonstiges (bitte Freitext am Ende der Seite nutzen)		vollkommen sicher	eher sicher	eher nicht sicher	überhaupt nicht sicher
○	○	○	○	○		○	○	○	○

Aussage 3: Bei der Gabe von **25 mg** Levomepromazin s.c. oder i.v. als Einzeldosis ist bei > 50% der o.g. „Standardpatient*innen" mit einer Sedierung von mind. -3 auf der RASS-PAL-Skala (mäßige Sedierung) zu rechnen. (Keine Daten zur oralen Gabe verfügbar. Aufgrund der geringen oralen Bioverfügbarkeit (20-50%) [1] ist von jeweils der doppelten Dosis für orale Gabe auszugehen.)

Grundlagen für diesen Vorschlag: Klinische Erfahrung und verfügbare Literatur (s. unten; u.a.:de Graeff et al. 2007: Median für „palliative Sedierung" (vermutlich tiefere Sedierung) 100 mg/24h).

Bitte wählen Sie die zutreffende Antwort für jeden Punkt aus:

Aussage 3 Levomepromazin						Wie sicher fühlen Sie sich in der Beantwortung der Frage?			
stimme vollkommen zu	stimme eher zu	stimme eher nicht zu	stimme überhaupt nicht zu	Sonstiges (bitte Freitext am Ende der Seite nutzen)		vollkommen sicher	eher sicher	eher nicht sicher	überhaupt nicht sicher
○	○	○	○	○		○	○	○	○

Freitextfeld für Ihre Kommentare:

Literatur:

[1]: Rémi, C., Bausewein, C., Charlesworth, S., Wilcock, A., & Howard, P. (Eds.). (2022). *Arzneimitteltherapie in der Palliativmedizin*. Elsevier Health Sciences.

[2]: European Association for Palliative Care (EAPC) recommended guideline for the use of sedation in palliative care (DRAFT)

- Palliative sedation (ohne Angabe der Sedierungstiefe):
  - Initialdosis: 12.5-25mg s.c. oder i.v.
  - Erhaltungsdosis: 0.5-8mg/h s.c. oder iv in Kombination mit Midazolam

[3]: Cherny, N. I., Radbruch, L., & Board of the European Association for Palliative Care. (2009). European Association for Palliative Care (EAPC) recommended framework for the use of sedation in palliative care. *Palliative medicine*, *23*(7), 581-593.

- Therapeutic / palliative sedation (ohne Angabe der Sedierungstiefe):
  - Bolusgabe 12.5-25mg, 50-75mg kontinuierlich i.v.
  - "Usual effective dose" 12.5-25mg/8Std sowie zusätzliche Bolusgaben wenn nötig.
  - Höchstdosis 300mg/Tag i.v.

[4]: KNMG (Hrsg.) (2009). Guideline for Palliative Sedation. Royal Dutch Medical Association (KNMG).

- Palliative sedation (ohne Angabe der Sedierungstiefe, aber vermutlich eher für tiefere Sedierung)
  - Bolus: 25 mg s.c./i.v., wenn nötig alle 2 Stunden 50 mg
  - Kontinuierlich: 0.5-8 mg/h s.c./i.v. (entspricht 12 – 192 mg/24 h) in Kombination mit 20 mg/h Midazolam. Nach drei Tagen Dosis halbieren.

[5]: de Graeff, A., & Dean, M. (2007). Palliative sedation therapy in the last weeks of life: a literature review and recommendations for standards. *Journal of palliative medicine*, *10*(1), 67–85.

- Palliative sedation (ohne Angabe der Sedierungstiefe, aber vermutlich eher für tiefere Sedierung)
  - In den letzten 48 Stunden: Median 100 mg/24h, Mittelwert 64 mg/24h
  - Spannweite: 25-250 mg/24h

[6]: Acedo Claro, C., & Rodríguez Martín, B. (2021). Sedación paliativa. *Revista Clínica de Medicina de Familia*, *14*(2), 93-97.

- Sedación paliativa (ohne Angabe einer Sedierungstiefe)
  - Beginn mit Bolusgaben 12.5-25mg alle 6 Stunden
  - Übliche wirksame Dosis 100mg/24h

[7]: Hardy, J. R., Skerman, H., Philip, J., Good, P., Currow, D. C., Mitchell, G., & Yates, P. (2019). Methotrimeprazine versus haloperidol in palliative care patients with cancer-related nausea: a randomised, double-blind controlled trial. *BMJ open*, *9*(9), e029942.

- Bei Anwendung von 6.25 oder 12.5mg/24 h p.o.: 10 von 49 Patient*innen (n= 15 Pat. (31%) hatte Dosis von 12,5 mg/h) berichten "Drowsiness"/Schläfrigkeit

[8]: Stone, P., Phillips, C., Spruyt, O., & Waight, C. (1997). A comparison of the use of sedatives in a hospital support team and in a hospice. *Palliative medicine*, *11*(2), 140–144.

- Retrospektive Aktenanalyse in 2 Einrichtungen (Hospiz und Palliativdienst im Krankenhaus)
- Bei Aktendurchsicht Klassifizierung des Gebrauchs des Medikaments als „für spezifische Symptomkontrolle“ oder „für Sedierung“
- Beispiel für Symptomkontrolle: Haloperidol als Antiemetikum
- Beispiel für Sedierung: Levomepromazine für eine agitierte, delirante Patient*in, die nicht behandelt werden konnte, ohne zu beabsichtigen, ihr Bewusstsein zu reduzieren.
- Angabe von 3 Mittelwerten für Medikamentendosis (nicht näher bezeichnet, vermutlich Tagesgesamtdosis): gesamt/Hospiz/Palliativdienst;
  keine Angabe von Spannweite oder Standardabweichung
- „Für Symptomkontrolle“: Mittelwert am Todestag: 58/38/100 mg/24h
- „Für Sedierung“ (cave: keine Angabe zu Sedierungstiefe):
  Mittelwert am Todestag: 64/52/81 mg/24h

[9]: Sykes, N., & Thorns, A. (2003). Sedative use in the last week of life and the implications for end-of-life decision making. *Archives of internal medicine*, *163*(3), 341–344.

- Retrospektive Aktenanalyse, bei der cut-off-Werte für potentiell sedierende Medikamente bestimmt wurden, oberhalb derer “signifikante Sedierung” “wahrscheinliche Folge” war
- Für Levompepromazin: Tagesgesamtdosis 25mg/24h

3.5 Haloperidol

Haloperidol

Aussage 1: Bei Haloperidol ist aufgrund der pharmakokinetischen Charakteristika der Substanz bei o.g. Standardpatient*innen ein anhaltender klinischer Effekt (nicht notwendigerweise Sedierung) zu erwarten, wenn es **alle 12 – 24 Stunden** angewendet wird.

Plasma-Halbwertszeit: 13 – 36 Std. [1]

Begründung für Vorschlag des Dosierintervalls für anhaltenden klinischen Effekt: Laut Fachinfo bei akuten psychomotischen Erregungszuständen 1 – 2 mal tgl. Gabe empfohlen. Wir gehen bei dieser Indikation von einem anhaltenden klinischen Effekt aus, der mit dem angegebenen Dosierintervall erzielt wird.

Bitte wählen Sie die zutreffende Antwort für jeden Punkt aus:

Aussage 1 Haloperidol						Wie sicher fühlen Sie sich in der Beantwortung der Frage?			
stimme vollkommen zu	stimme eher zu	stimme eher nicht zu	stimme überhaupt nicht zu	Sonstiges (bitte Freitext am Ende der Seite nutzen)		vollkommen sicher	eher sicher	eher nicht sicher	überhaupt nicht sicher
○	○	○	○	○		○	○	○	○

Aussage 2: Bei der Gabe von **4 mg** Haloperidol s.c. oder i.v. als Einzeldosis ist bei > 50% der o.g. „Standardpatient*innen“ mit einer Sedierung von mind. -1 auf der RASS-PAL-Skala (leichte Sedierung) zu rechnen. (Keine Daten zur oralen Gabe verfügbar. Aufgrund der sehr variablen Bioverfügbarkeit (zwischen 45 und 85% [2]) setzen wir die cut-off-Werte für die orale Gabe gleich an.)

Grundlagen für diesen Vorschlag: klinische Erfahrung und verfügbare Literatur (s. unten)

Bitte wählen Sie die zutreffende Antwort für jeden Punkt aus:

**Aussage 2 Haloperidol**						**Wie sicher fühlen Sie sich in der Beantwortung der Frage?**			
**stimme vollkommen zu**	**stimme eher zu**	**stimme eher nicht zu**	**stimme überhaupt nicht zu**	**Sonstiges (bitte Freitext am Ende der Seite nutzen)**		**vollkommen sicher**	**eher sicher**	**eher nicht sicher**	**überhaupt nicht sicher**
○	○	○	○	○		○	○	○	○

Aussage 3: Wir schlagen **keinen cut-off-Wert** für Haloperidol vor, bei dem bei > 50% der o.g. „Standardpatient*innen“ mit einer Sedierung von mind. -3 auf der RASS-PAL-Skala (mäßige Sedierung) zu rechnen ist, aufgrund von großer Variabilität des individuellen sedierenden Effektes.

Grundlagen für diesen Vorschlag: Klinische Erfahrung und Literatur (s. unten).

Bitte wählen Sie die zutreffende Antwort für jeden Punkt aus:

Aussage 3 Haloperidol						Wie sicher fühlen Sie sich in der Beantwortung der Frage?			
stimme vollkommen zu	stimme eher zu	stimme eher nicht zu	stimme überhaupt nicht zu	Sonstiges (bitte Freitext am Ende der Seite nutzen)		vollkommen sicher	eher sicher	eher nicht sicher	überhaupt nicht sicher
○	○	○	○	○		○	○	○	○

Wenn Sie einen Vorschlag für einen cut-off-Wert haben, schreiben Sie ihn bitte inklusive Begründung und ggf. Literatur in das Freitextfeld.

Freitextfeld für Ihre Kommentare:

Literatur:

[1]: Fachinfo Haldol Janssen Injektionslösung, Stand 09/2019

[2]: Rémi, C., Bausewein, C., Charlesworth, S., Wilcock, A., & Howard, P. (Eds.). (2022). *Arzneimitteltherapie in der Palliativmedizin*. Elsevier Health Sciences.

[3]: Stone, P., Phillips, C., Spruyt, O., & Waight, C. (1997). A comparison of the use of sedatives in a hospital support team and in a hospice. *Palliative medicine*, *11*(2), 140–144.

- retrospektive Aktenanalyse in 2 Einrichtungen (Hospiz und Palliativdienst im Krankenhaus),
- Bei Aktendurchsicht Klassifizierung des Gebrauchs des Medikaments als „für spezifische Symptomkontrolle“ oder „für Sedierung“.
- Beispiel für Symptomkontrolle: Haloperidol als Antiemetikum,
- Beispiel für Sedierung: Levomepromazine für eine agitierte, delirante Patient*in, die nicht behandelt werden konnte, ohne zu beabsichtigen, ihr Bewusstsein zu reduzieren.
- Angabe von 3 Mittelwerten für Medikamentendosis (nicht näher bezeichnet, vermutlich Tagesgesamtdosis): gesamt/Hospiz/Palliativdienst;
  keine Angabe von Spannweite oder Standardabweichung
- „Für Symptomkontrolle“: Mittelwert am Todestag: 6/6/7 mg/24 h
- „Für Sedierung“ (cave: keine Angabe zu Sedierungstiefe):
  Mittelwert am Todestag: 5/4/7 mg/24 h
- Zusätzlich unterstützend bzgl. der Einschätzung “bei < 4 mg nicht bei > 50% der Pat. sedierender Effekt“.

[5]: Sykes, N., & Thorns, A. (2003). Sedative use in the last week of life and the implications for end-of-life decision making. *Archives of internal medicine*, *163*(3), 341–344.

- Retrospective Aktenanalyse, bei der cut-off-Werte für potentiell sedierende Medikamente bestimmt wurden, oberhalb derer “signifikante Sedierung” “wahrscheinliche Folge” war
- Für Haloperidol: Tagesgesamtdosis 20mg/24 h

3.6 Melperon

Melperon

Aussage 1: Bei Melperon ist aufgrund der pharmakokinetischen Charakteristika der Substanz bei o.g. Standardpatient*innen ein anhaltender klinischer Effekt (nicht notwendigerweise Sedierung) zu erwarten, wenn es **alle 6 – 12 Stunden** angewendet wird.

Plasma-Halbwertszeit: 4 – 6 Std. [1, 2]

Begründung für Vorschlag des Dosierintervalls für anhaltenden klinischen Effekt: Laut Fachfinfo und Benkert-Hippius [2] bei psychomotorischen Erregungszuständen 2 – 4 mal tgl. Gabe empfohlen. Wir gehen bei dieser Indikation von einem anhaltenden klinischen Effekt aus, der mit dem angegebenen Dosierintervall erzielt wird.

Bitte wählen Sie die zutreffende Antwort für jeden Punkt aus:

Aussage 1 Melperon						Wie sicher fühlen Sie sich in der Beantwortung der Frage?			
stimme vollkommen zu	stimme eher zu	stimme eher nicht zu	stimme überhaupt nicht zu	Sonstiges (bitte Freitext am Ende der Seite nutzen)		vollkommen sicher	eher sicher	eher nicht sicher	überhaupt nicht sicher
○	○	○	○	○		○	○	○	○

Aussage 2: Bei der Gabe von **50 mg** Melperon oral als Einzeldosis ist bei > 50% der o.g. „Standard-patient*innen" mit einer Sedierung von mind. -1 auf der RASS-PAL-Skala (leichte Sedierung) zu rechnen.

Grundlagen für diesen Vorschlag: Dosierungsempfehlungen für Schlafinduktion (siehe Literatur unten), klinische Erfahrung

Bitte wählen Sie die zutreffende Antwort für jeden Punkt aus:

Aussage 2 Melperon						Wie sicher fühlen Sie sich in der Beant-wortung der Frage?			
stimme voll-kom-men zu	stimme eher zu	stimme eher nicht zu	stimme über-haupt nicht zu	Sonsti-ges (bitte Freitext am Ende der Sei-te nutzen)		voll-kom-men sicher	eher sicher	eher nicht sicher	über-haupt nicht sicher
○	○	○	○	○		○	○	○	○

Aussage 3: Bei der Gabe von **100 mg** Melperon oral als Einzeldosis ist bei > 50% der o.g. „Standard-patient*innen“ mit einer Sedierung von mind. -3 auf der RASS-PAL-Skala (mäßige Sedierung) zu rechnen.

Grundlagen für diesen Vorschlag: Dosierungsempfehlungen für Schlafinduktion (siehe Literatur unten), klinische Erfahrung

Bitte wählen Sie die zutreffende Antwort für jeden Punkt aus:

Aussage 3 Melperon						Wie sicher fühlen Sie sich in der Beantwortung der Frage?			
stimme vollkommen zu	stimme eher zu	stimme eher nicht zu	stimme überhaupt nicht zu	Sonstiges (bitte Freitext am Ende der Seite nutzen)		vollkommen sicher	eher sicher	eher nicht sicher	überhaupt nicht sicher
○	○	○	○	○		○	○	○	○

Freitextfeld für Ihre Kommentare:

Literatur:

[1]: Fachinfo Melperon neuraxpharm Stand 03/2015:

- allgemeiner Dosisbereich 25 – 400 mg, Anpassung an individuelle Reaktionslage, Alter und Gewicht des Patienten sowie Art und Schwere des Krankheitsbildes notwendig

[2]: Benkert, O., Hippius, H., & GmbH, S. (2019). *Kompendium der Psychiatrischen Pharmakotherapie* (12., vollständig überarbeitete und aktualisierte Auflage.). Springer.

[3]: Rémi, C., Bausewein, C., Charlesworth, S., Wilcock, A., & Howard, P. (Eds.). (2022). *Arzneimitteltherapie in der Palliativmedizin*. Elsevier Health Sciences.

- Dosierungsempfehlungen für Schlafinduktion: 25-200 mg

[4]: Leitlinienprogramm Onkologie (2021). Erweiterte S3-Leitlinie Palliativmedizin für Patienten mit einer nicht heilbaren Krebserkrankung, Kurzversion 2.3

- Insomnie 10-100mg

3.7 Pipamperon

Pipamperon

Aussage 1: Bei Pipamperon ist aufgrund der pharmakokinetischen Charakteristika der Substanz bei o.g. Standardpatient*innen ein anhaltender klinischer Effekt (nicht notwendigerweise Sedierung) zu erwarten, wenn es **alle 8 Stunden** angewendet wird.

Plasma-Halbwertszeit: 17 – 22 Std. [1]

Begründung für Vorschlag des Dosierintervalls für anhaltenden klinischen Effekt: Lt. Fachinfo bei psychomotorischen Erregungszuständen 3 x tgl. Gabe empfohlen. Wir gehen bei dieser Indikation von einem anhaltenden klinischen Effekt aus, der mit dem angegebenen Dosierintervall erzielt wird.

Bitte wählen Sie die zutreffende Antwort für jeden Punkt aus:

Aussage 1 Pipamperon						Wie sicher fühlen Sie sich in der Beantwortung der Frage?			
stimme vollkommen zu	stimme eher zu	stimme eher nicht zu	stimme überhaupt nicht zu	Sonstiges (bitte Freitext am Ende der Seite nutzen)		vollkommen sicher	eher sicher	eher nicht sicher	überhaupt nicht sicher
○	○	○	○	○		○	○	○	○

Aussage 2: Bei der Gabe von **40 mg** Pipamperon oral als Einzeldosis ist bei > 50% der o.g. „Standardpatient*innen" mit einer Sedierung von mind. -1 auf der RASS-PAL-Skala (leichte Sedierung) zu rechnen.

Grundlagen für diesen Vorschlag: Dosierungsempfehlungen für Schlafinduktion (siehe Literatur unten) und klinische Erfahrung.

Bitte wählen Sie die zutreffende Antwort für jeden Punkt aus:

Aussage 2 Pipamperon						Wie sicher fühlen Sie sich in der Beantwortung der Frage?			
stimme vollkommen zu	stimme eher zu	stimme eher nicht zu	stimme überhaupt nicht zu	Sonstiges (bitte Freitext am Ende der Seite nutzen)		vollkommen sicher	eher sicher	eher nicht sicher	überhaupt nicht sicher
○	○	○	○	○		○	○	○	○

Aussage 3: Bei der Gabe von **120 mg** Pipamperon oral als Einzeldosis ist bei > 50% der o.g. „Standardpatient*innen“ mit einer Sedierung von mind. -3 auf der RASS-PAL-Skala (mäßige Sedierung) zu rechnen.

Grundlagen für diesen Vorschlag: Dosierungsempfehlungen für Schlafinduktion (siehe Literatur unten) und klinische Erfahrung.

Bitte wählen Sie die zutreffende Antwort für jeden Punkt aus:

Aussage 3 Pipamperon						Wie sicher fühlen Sie sich in der Beantwortung der Frage?			
stimme vollkommen zu	stimme eher zu	stimme eher nicht zu	stimme überhaupt nicht zu	Sonstiges (bitte Freitext am Ende der Seite nutzen)		vollkommen sicher	eher sicher	eher nicht sicher	überhaupt nicht sicher
○	○	○	○	○		○	○	○	○

Freitextfeld für Ihre Kommentare:

Literatur:

[1]: Fachinfo Pipamperon- neuraxpharm®, Stand 04/2018:

- Schlafstörung: 40 mg

[2]: Leitlinienprogramm Onkologie (2021). Erweiterte S3-Leitlinie Palliativmedizin für Patienten mit einer nicht heilbaren Krebserkrankung, Kurzversion 2.3

- Insomnie 40-120mg

[3]: Benkert, O., Hippius, H., & GmbH, S. (2019). *Kompendium der Psychiatrischen Pharmakotherapie* (12., vollständig überarbeitete und aktualisierte Auflage.). Springer.

- Indikation: Schlafstörungen, insbesondere bei geriatrischen Patienten, psychomotorische Erregungszustände
    - Dosis: 40–120 mg; nicht mehr als 360 mg/Tag

3.8 Phenobarbital

Phenobarbital

Aussage 1: Bei Phenobarbital ist aufgrund der pharmakokinetischen Charakteristika der Substanz bei o.g. Standardpatient*innen ein anhaltender klinischer Effekt (nicht notwendigerweise Sedierung) zu erwarten, wenn es **alle 24 Stunden** angewendet wird.

Plasma-Halbwertszeit: 75 – 120 Std. [1]

Begründung für Vorschlag des Dosierintervalls für anhaltenden klinischen Effekt: Nach dem persönlichen Ermessen des Studienteams aufgrund der langen Halbwertszeit, obwohl lt. Fachinfo [1] zur Epilepsie-Behandlung mehrmals tägliche Applikation empfohlen wird.

Bitte wählen Sie die zutreffende Antwort für jeden Punkt aus:

Aussage 1 Phenobarbital						Wie sicher fühlen Sie sich in der Beantwortung der Frage?			
stimme vollkommen zu	stimme eher zu	stimme eher nicht zu	stimme überhaupt nicht zu	Sonstiges (bitte Freitext am Ende der Seite nutzen)		vollkommen sicher	eher sicher	eher nicht sicher	überhaupt nicht sicher
○	○	○	○	○		○	○	○	○

Aussage 2: Bei der Gabe von **100 mg** Phenobarbital i.v. als Einzeldosis ist bei > 50% der o.g. „Standardpatient*innen“ mit einer Sedierung von mind. -1 auf der RASS-PAL-Skala (leichte Sedierung) zu rechnen.

Grundlagen für diesen Vorschlag: Klinische Einschätzung auf Grundlage der verfügbaren Literatur (s. unten).

Bitte wählen Sie die zutreffende Antwort für jeden Punkt aus:

Aussage 2 Phenobarbital						Wie sicher fühlen Sie sich in der Beantwortung der Frage?			
stimme vollkommen zu	stimme eher zu	stimme eher nicht zu	stimme überhaupt nicht zu	Sonstiges (bitte Freitext am Ende der Seite nutzen)		vollkommen sicher	eher sicher	eher nicht sicher	überhaupt nicht sicher
○	○	○	○	○		○	○	○	○

Aussage 3: Bei der Gabe von **400 mg** Phenobarbital i.v. als Einzeldosis ist bei > 50% der o.g. „Standardpatient*innen" mit einer Sedierung von mind. -3 auf der RASS-PAL-Skala (mäßige Sedierung) zu rechnen.

Grundlagen für diesen Vorschlag: Grundlage für diesen Vorschlag: klinische Einschätzung auf Grundlage der verfügbaren Literatur (s. unten).

Bitte wählen Sie die zutreffende Antwort für jeden Punkt aus:

Aussage 3 Phenobarbital						Wie sicher fühlen Sie sich in der Beantwortung der Frage?			
stimme vollkommen zu	stimme eher zu	stimme eher nicht zu	stimme überhaupt nicht zu	Sonstiges (bitte Freitext am Ende der Seite nutzen)		vollkommen sicher	eher sicher	eher nicht sicher	überhaupt nicht sicher
○	○	○	○	○		○	○	○	○

Freitextfeld für Ihre Kommentare:

Literatur:

[1]: Fachinfo Luminal Injektionslösung, Stand 08/2021

[2]: de Graeff, A., & Dean, M. (2007). Palliative sedation therapy in the last weeks of life: a literature review and recommendations for standards. *Journal of palliative medicine*, *10*(1), 67–85.

- Palliative sedation (ohne Angabe der Sedierungstiefe, aber vermutlich eher für tiefere Sedierung)
    - In den letzten 48 Stunden:
        - Median: 800-1600 mg/24h
        - Spannweite: 200-2500 mg/24h

[3]: Won, Y. W., Chun, H. S., Seo, M., Kim, R. B., Kim, J. H., & Kang, J. H. (2019). Clinical Patterns of Continuous and Intermittent Palliative Sedation in Patients With Terminal Cancer: A Descriptive, Observational Study. *Journal of pain and symptom management*, *58*(1), 65–71.

- (n=3 Pat. Erhielten Phenobarbital)
    - Bolus bei Start: 100 – 200 mg i.v.
    - Erhaltungsdosis 25 – 50 mg/h i.v.

[4]: Cheng, C., Roemer-Becuwe, C., & Pereira, J. (2002). When midazolam fails. *Journal of pain and symptom management*, *23*(3), 256–265.

- Dosisempfehlungen auf Basis von 2 Literaturreferenzen
    - Startdosis: 100-200mg Bolus i.m.
    - kontinuierliche Dosis zwischen 200 und 2500mg/24h (s.c.) (Median: 1200mg/24h)
    - "Sedation in a catastrophic event": 100-200mg i.m. oder s.c. Bolus

3.9 Propofol

Propofol

Aussage 1: Bei Propofol ist aufgrund der pharmakokinetischen Charakteristika der Substanz bei o.g. Standardpatient*innen ein anhaltender klinischer Effekt (nicht notwendigerweise Sedierung) bei **kontinuierlicher Gabe** zu erwarten.

Plasma-Halbwertszeit: Je nach Verteilungsphase 1,8 - 4,1 min bzw. 34 – 64 min. [1]

Begründung für Vorschlag des Dosierintervalls für anhaltenden klinischen Effekt: Lt. Fachinfo nur sehr kurze Wirkdauer. Wir gehen davon aus, dass ein anhaltender klinischer Effekt in aller Regel nur bei kontinuierlicher Applikation erzielt wird.

Bitte wählen Sie die zutreffende Antwort für jeden Punkt aus:

Aussage 1 Propofol						Wie sicher fühlen Sie sich in der Beantwortung der Frage?			
stimme vollkommen zu	stimme eher zu	stimme eher nicht zu	stimme überhaupt nicht zu	Sonstiges (bitte Freitext am Ende der Seite nutzen)		vollkommen sicher	eher sicher	eher nicht sicher	überhaupt nicht sicher
○	○	○	○	○		○	○	○	○

Aussage 2: Bei der Gabe von **0,1 mg/kg KG/h** Propofol i.v. ist bei > 50% der o.g. „Standardpatient*innen“ mit einer Sedierung von mind. -1 auf der RASS-PAL-Skala (leichte Sedierung) zu rechnen.

Grundlagen für diesen Vorschlag: Klinische Einschätzung auf Grundlage der Literatur (s. unten, v.a.: Empfehlung von 0,3 mg/kg KG/h für Sedierung im Rahmen der Intensivbehandlung (Fachinfo) und 0,3 mg/kg KG/h als Startdosis für „palliative Sedierung“ (KNMG-Leitlinie)) und klinischer Erfahrung).

Bitte wählen Sie die zutreffende Antwort für jeden Punkt aus:

Aussage 2 Propofol						Wie sicher fühlen Sie sich in der Beantwortung der Frage?			
stimme vollkommen zu	stimme eher zu	stimme eher nicht zu	stimme überhaupt nicht zu	Sonstiges (bitte Freitext am Ende der Seite nutzen)		vollkommen sicher	eher sicher	eher nicht sicher	überhaupt nicht sicher
○	○	○	○	○		○	○	○	○

Aussage 3: Bei Propofol ist bei jeder weiteren Dosiserhöhung, ausgehend von **0,1 mg/kg KG/h**, bei > 50% der o.g. „Standardpatient*innen" mit einer Sedierung von mind. -3 auf der RASS-PAL-Skala (mäßige Sedierung) zu rechnen. (Keine Festlegung eines cut-off-Wertes).

Grundlagen für diesen Vorschlag: Klinische Erfahrung.

Bitte wählen Sie die zutreffende Antwort für jeden Punkt aus:

Aussage 3 Propofol						Wie sicher fühlen Sie sich in der Beantwortung der Frage?			
stimme vollkommen zu	stimme eher zu	stimme eher nicht zu	stimme überhaupt nicht zu	Sonstiges (bitte Freitext am Ende der Seite nutzen)		vollkommen sicher	eher sicher	eher nicht sicher	überhaupt nicht sicher
○	○	○	○	○		○	○	○	○

Wenn Sie einen Vorschlag für einen cut-off-Wert haben, schreiben Sie ihn bitte inklusive Begründung und ggf. Literatur in das Freitextfeld.

Freitextfeld für Ihre Kommentare:

Literatur:

[1]: Fresenius Kabi (2021): Fachinfo Propofol Disoprivan 1% (10 mg/1 ml) MCT Fresenius, Emulsion zur Injektion oder Infusion, Amp. 20ml

- Sedierung von Erwachsenen bei chirurgischen und diagnostischen Maßnahmen:
  - Zur Einleitung meistens 0.5-1mg/kg über 1-5 min
  - Aufrechterhaltung zwischen 1.5-4.5mg/kg/h
  - Zusätzliche Bolusgabe 10-20mg möglich
- Sedierung von Patienten über 16 Jahre im Rahmen der Intensivbehandlung:
  - Übliche Infusionsrate zwischen 0.3 bis 4mg/kg/h
  - 4mg/kg/h sollte nicht überschritten werden
- Allgemeinanästhesie bei Erwachsenen:
  - Einleitung: 1.5-2.5mg/kg, bei Pat über 55 Jahre oder schlechtem AGZ 1mg/kg
  - Aufrechterhaltungsdosis üblicherweise zwischen 4-12mg/kg/h

[2]: European Association for Palliative Care (EAPC) recommended guideline for the use of sedation in palliative care (DRAFT)

- "Palliative sedation" (im Unterschied zu Midazolam hier keine unterschiedlichen Dosierungs-Angaben für leichte oder tiefe „palliative sedation")
  - Initialdosis: 1mg/kg/h, alle 30 min um 0.5mg/kg/h erhöhen, bis gewünschte Sedierungstiefe erreicht.

[3]: KNMG (2009). Guideline for Palliative Sedation. Royal Dutch Medical Association (KNMG).

- ("Palliative sedation" nicht näher definiert, aber vermutlich als "tiefe" Sedierung gemeint):
  - Bolus 20-50 mg i.v. für kurze Sedierung
  - 20 mg/h i.v. (entspricht 0,3 mg/kg KG/h bei 70 kg KG), Erhöhung um 10 mg/h alle 10 min, bis gewünschte Sedierungstiefe erreicht

[4]: NCCN (2016): NCCN Clinical Practice Guidelines in Oncology (NCCN Guidelines). Palliative Care (Präsentation).

- Für „Palliative sedation" bei „unmittelbar sterbenden" Patient*innen, Sedierungstiefe nicht näher definiert
  - Startdosis 5-10 µg/kg/min (entspricht 0,35 – 0,7 mg/kg/h)

[5]: Acedo Claro, C., & Rodríguez Martín, B. (2021). Sedación paliativa. *Revista Clínica de Medicina de Familia*, *14*(2), 93-97.

- Sedación paliativa:
  - Startdosis: 0.5-1mg/kg/h
  - Dosissteigerung um 0.25-0.5mg/kg/h alle 5-10 min
  - Übliche effektive Dosis: 1-2mg/kg/h

3.10 Dexmedetomidin

Dexmedetomidin

Aussage 1: Bei Dexmedetomidin ist aufgrund der pharmakokinetischen Charakteristika der Substanz bei o.g. Standardpatient*innen ein anhaltender klinischer Effekt (nicht notwendigerweise Sedierung) bei **kontinuierlicher Gabe** zu erwarten.

Plasma-Halbwertszeit: 1,9 – 2,5 Std. [1]

Begründung für Vorschlag des Dosierintervalls für anhaltenden klinischen Effekt: Aufgrund kurzer Wirkdauer in aller Regel nur kontinuierliche Applikation.

Bitte wählen Sie die zutreffende Antwort für jeden Punkt aus:

Aussage 1 Dexmedetomidin						Wie sicher fühlen Sie sich in der Beantwortung der Frage?			
stimme vollkommen zu	stimme eher zu	stimme eher nicht zu	stimme überhaupt nicht zu	Sonstiges (bitte Freitext am Ende der Seite nutzen)		vollkommen sicher	eher sicher	eher nicht sicher	überhaupt nicht sicher
○	○	○	○	○		○	○	○	○

ANSICHT DER NUTZER:INNEN

Aussage 2: Bei der Gabe von **0,4 µg/kg/h** Dexmedetomidin i.v. ist bei > 50% der o.g. „Standardpatient*innen“ mit einer Sedierung von mind. -1 auf der RASS-PAL-Skala (leichte Sedierung) zu rechnen.

Grundlagen für diesen Vorschlag: Herstellerempfehlung, weitere Literatur (s. unten) und klinische Erfahrung.

Bitte wählen Sie die zutreffende Antwort für jeden Punkt aus:

Aussage 2 Dexmedetomidin						Wie sicher fühlen Sie sich in der Beantwortung der Frage?			
stimme vollkommen zu	stimme eher zu	stimme eher nicht zu	stimme überhaupt nicht zu	Sonstiges (bitte Freitext am Ende der Seite nutzen)		vollkommen sicher	eher sicher	eher nicht sicher	überhaupt nicht sicher
○	○	○	○	○		○	○	○	○

Aussage 3: Bei der Gabe von **0,6 µg/kg/h** Dexmedetomidin i.v. ist bei > 50% der o.g. „Standardpatient*innen“ mit einer Sedierung von mind. -3 auf der RASS-PAL-Skala (mäßige Sedierung) zu rechnen.

Grundlagen für diesen Vorschlag: Herstellerempfehlung, weitere Literatur (s. unten) und klinische Erfahrung.

Bitte wählen Sie die zutreffende Antwort für jeden Punkt aus:

**Aussage 3 Dexmedetomidin**						**Wie sicher fühlen Sie sich in der Beantwortung der Frage?**			
**stimme vollkommen zu**	**stimme eher zu**	**stimme eher nicht zu**	**stimme überhaupt nicht zu**	**Sonstiges (bitte Freitext am Ende der Seite nutzen)**		**vollkommen sicher**	**eher sicher**	**eher nicht sicher**	**überhaupt nicht sicher**
○	○	○	○	○		○	○	○	○

Freitextfeld für Ihre Kommentare:

Literatur:

[1]: Fachinfo Dexdor 100 Mikrogramm/ml Konzentrat zur Herstellung einer Infusionslösung, Ampulle 2ml, Orion Pharma (Stand 2020).

- "Für die Sedierung erwachsener, intensivmedizinisch behandelter Patienten," die eine leichte(re) Sed (RASS zwischen 0 und -3) benötigen:
  Anfangsdosis 0.7 µg/kg/h, übliche Dosis zwischen 0.2 und 1.4 µg/kg/h. Anpassung der Dosis stündlich. Höchstdosis: 1.4 µg/kg/h.
- Für prozedurale Sedierung/Wachsedierung:
  0,1 µg/kg für 10 Minuten, Erhaltungsdosis wie oben.

[2]: Keating, G. M. (2015). Dexmedetomidine: A Review of Its Use for Sedation in the Intensive Care Setting. *Drugs*, *75*(10), 1119–1130.

- "The usual goal of sedation in the intensive care unit (ICU) is a calm, cooperative patient who is easy to rouse and who is able to communicate their needs, particularly for analgesia"
- Longer term sedation (>=24h), angestrebtes Ziel: RASS 0 - -3:
  Keine Info zu loading dose, maintenance dose 0.2-1.4 µg/kg/h

[3]: Thomas, B., Lo, W. A., Nangati, Z., & Barclay, G. (2021). Dexmedetomidine for hyperactive delirium at the end of life: An open-label single arm pilot study with dose escalation in adult patients admitted to an inpatient palliative care unit. *Palliative medicine*, *35*(4), 729–737.

- Ziel: Memorial Delirium Assessment Scale (MDAS) < 13, RASS-PAL -1 bis -3
  2 Dosisstufen: 0.3 oder 0.6 µg/kg/h
  n=22
- n=13 benötigten Dosiseskalation auf Dosis-Stufe 2 (0,6 µg/kg/h) für die Kontrolle des Delirs
  RASS-PAL-Werte: große Variabilität, aber RASS-PAL-Mittelwerte lagen für beide Dosisstufen jeweils im Zielbereich (- 1 bis -3)

3.11 Trazodon

Trazodon

Aussage 1: Bei Trazodon ist aufgrund der pharmakokinetischen Charakteristika der Substanz bei o.g. Standardpatient*innen ein anhaltender klinischer Effekt (nicht notwendigerweise Sedierung) zu erwarten, wenn es **alle 12 Stunden** angewendet wird.

Plasma-Halbwertszeit: 5 – 8 Std. [1, 2]

Begründung für Vorschlag des Dosierintervalls für anhaltenden klinischen Effekt: Lt. Fachinfo 1 – 2 mal tgl. Gabe als Antidepressivum, auf Basis der Halbwertszeit und Sedierung lediglich als Nebenwirkung der Substanz haben wir für die Bestimmung eines anhaltenden klinischen Effekts das kürzere Applikationsintervall herangezogen.

Bitte wählen Sie die zutreffende Antwort für jeden Punkt aus:

Aussage 1 Trazodon						Wie sicher fühlen Sie sich in der Beantwortung der Frage?			
stimme vollkommen zu	stimme eher zu	stimme eher nicht zu	stimme überhaupt nicht zu	Sonstiges (bitte Freitext am Ende der Seite nutzen)		vollkommen sicher	eher sicher	eher nicht sicher	überhaupt nicht sicher
○	○	○	○	○		○	○	○	○

Aussage 2: Bei der Gabe von **50 mg** Trazodon oral als Einzeldosis ist bei > 50% der o.g. „Standard-patient*innen“ mit einer Sedierung von mind. -1 auf der RASS-PAL-Skala (leichte Sedierung) zu rechnen.

Grundlagen für diesen Vorschlag: Dosierungsempfehlungen für Schlafstörungen (siehe Literatur unten).

Bitte wählen Sie die zutreffende Antwort für jeden Punkt aus:

Aussage 2 Trazodon						Wie sicher fühlen Sie sich in der Beantwortung der Frage?			
stimme vollkommen zu	stimme eher zu	stimme eher nicht zu	stimme überhaupt nicht zu	Sonstiges (bitte Freitext am Ende der Seite nutzen)		vollkommen sicher	eher sicher	eher nicht sicher	überhaupt nicht sicher
○	○	○	○	○		○	○	○	○

Aussage 3: Wir schlagen für Trazodon **keinen cut-off-Wert** vor, bei dem bei > 50% der o.g. „Standardpatient*innen" mit einer Sedierung von mind. -3 auf der RASS-PAL-Skala (mäßige Sedierung) zu rechnen ist, aufgrund von zu wenig Daten und zu wenig klinischer Erfahrung.

Grundlagen für diesen Vorschlag: Klinische Erfahrung und Literatur (s. unten).

Bitte wählen Sie die zutreffende Antwort für jeden Punkt aus:

Aussage 3 Trazodon						Wie sicher fühlen Sie sich in der Beantwortung der Frage?			
stimme vollkommen zu	stimme eher zu	stimme eher nicht zu	stimme überhaupt nicht zu	sonstiges (bitte Freitext am Ende der Seite nutzen)		vollkommen sicher	eher sicher	eher nicht sicher	überhaupt nicht sicher
○	○	○	○	○		○	○	○	○

Wenn Sie einen Vorschlag für einen cut-off-Wert haben, schreiben Sie ihn bitte inklusive Begründung und ggf. Literatur in das Freitextfeld.

Freitextfeld für Ihre Kommentare:

Literatur:

[1]: Fachinfo Trazodon neuraxpharm, Stand 02/2015.

[2]: Benkert, O., Hippius, H., & GmbH, S. (2021). *Kompendium der Psychiatrischen Pharmakotherapie* (13. Auflage.). Springer.

- "Antidepressiva mit sedierenden Eigenschaften [...] wirken schlaffördernd und haben einen festen Platz in der Insomniebehandlung. Die empfohlenen abendlichen Dosierungen bei Insomnie (ohne depressive Störung oder Angststörung) sind: [...] Trazodon (25–150 mg)"

[3]: Tanimukai, H., Murai, T., Okazaki, N., Matsuda, Y., Okamoto, Y., Kabeshita, Y., Ohno, Y., & Tsuneto, S. (2013). An observational study of insomnia and nightmare treated with trazodone in patients with advanced cancer. *The American journal of hospice & palliative care*, *30*(4), 359–362.

- Regime bei Schlaflosigkeit:
  - Startdosis: 12,5 bis 25 mg, Dosiserhöhung auf 25 bis 50 mg, bis sich die Schlaflosigkeit verbesserte;
  - Meiste Pat. Dosis von 25 mg erhalten, hierdurch Verbesserung des Schlafs, aber keine „tiefere Sedierung“ (nicht näher definiert)

[4]: Kolla, B. P., Mansukhani, M. P., & Schneekloth, T. (2011). Pharmacological treatment of insomnia in alcohol recovery: a systematic review. *Alcohol and alcoholism (Oxford, Oxfordshire)*, *46*(5), 578–585.

- Review, fasst 2 Studien zu Therapie von Schlafstörungen bei Patient*innen mit Alkoholabhängigkeit nach Entzug zusammen:
  - Studie 1: Startdosis 50 mg, schrittweise erhöht auf 200 mg (teilweise nur 150 mg von Patient*innen toleriert)
  - Studie 2: Dosis von 50 – 150 mg (im Durchschnitt 100 mg) über 12 Wochen
  - In beiden Studien Verbesserung des Schlafs und anderer Endpunkte

[5]: Mehnert, A., & Breitbart, W. (2006). Diagnostik und Behandlung psychischer Störungen in der Palliativmedizin. *Koch U, Lang Mehnert A, Schmeling-Kludas (Hrsg) Die Begleitung schwer kranker und sterbender Menschen. Grundlagen und Anwendungshilfen in der Palliativversorgung. Schattauer, Stuttgart*, 90-122.

- Bei depressiven Patient*innen mit Schlafstörungen 50-100mg Trazodon

[6]: Leitlinienprogramm Onkologie (2021). Erweiterte S3-Leitlinie Palliativmedizin für Patienten mit einer nicht heilbaren Krebserkrankung, Kurzversion 2.3

- Insomnie (off label), 25-100mg

[7]: Palliative Care Formulary über https://about.medicinescomplete.com/; Zugriff am 10.2.2022

- Übliche effektive Dosis bei Insomnie: 50 mg zur Nacht

4. Weitere sedierende Medikamente

Weitere sedierende Medikamente

Bei allen Benzodiazepinen und verschiedenen Antipsychotika und Antidepressiva muss grundsätzlich die Möglichkeit einer sedierenden Wirkung mitberücksichtigt werden.

Gibt es aus Ihrer Sicht zusätzlich zu den in dieser Befragung aufgeführten Medikamenten noch weitere Substanzen, bei denen Sie ein relevantes Risiko sehen, dass im Rahmen der Anwendung dieser Substanz der Übergang zu einer Sedierung fließend sein kann, für die wir also auch solche cut-off-Werte bestimmen sollten?

Wenn ja, schreiben Sie die Substanz und ggf. Ihre Anmerkung/Begründung in das Freitextfeld.

(Anmerkung: Für weitere Vertreter dieser Substanzgruppen, die nicht in dieser Befragung genannt sind, gibt es keine oder sehr wenig Evidenz, die für solche cut-off-Werte herangezogen werden könnte. Die Bestimmung von cut-off-Werten für diese Substanzen würde also im Wesentlichen auf klinischer Erfahrung/Expert*innenmeinung beruhen.)

5. Zusätzliche Aspekte für das Gruppen-Delphi

Zusätzliche Aspekte für das Gruppen-Delphi

Gibt es sonst etwas, das Sie noch anmerken wollen, z.B. Thema in Zusammenhang mit dieser Befragung, das auf jeden Fall im geplanten Gruppen-Delphi-Verfahren diskutiert werden sollte?

6. Demographische Daten

Demographische Daten

Zum Abschluss unserer Befragung würden wir Sie noch um ein paar wenige Angaben zu Ihrer Person bitten.

Mit welchem Geschlecht identifizieren Sie sich?

Bitte wählen Sie eine der folgenden Antworten:

- ○ männlich
- ○ weiblich
- ○ andere: ____

Welcher Altersgruppe sind Sie zugehörig?

Bitte wählen Sie eine der folgenden Antworten:

- ○ unter 30 Jahre
- ○ 30-39 Jahre
- ○ 40-49 Jahre
- ○ 50-59 Jahre
- ○ 60 Jahre oder älter

Welcher Berufsgruppe sind Sie zugehörig?

Bitte wählen Sie eine der folgenden Antworten:

- ○ Ärzt*in
- ○ Pfleger*in
- ○ Pharmazeut*in

In welchem dieser Tätigkeitsbereiche arbeiten Sie aktuell?
(Mehrfachantworten möglich)

Bitte wählen Sie die zutreffenden Antworten aus:

- ☐ Allgemeinmedizin
- ☐ Anästhesie
- ☐ Innere Medizin
- ☐ Intensivmedizin
- ☐ Neurologie
- ☐ Palliativversorgung: Palliativdienst im Krankenhaus
- ☐ Palliativversorgung: Palliativstation
- ☐ Palliativversorgung: SAPV
- ☐ Andere: ____

Über welche Berufserfahrung verfügen Sie?
(Mehrfachantworten möglich)

Bitte wählen Sie die zutreffenden Antworten aus:

- [ ] über ein Jahr Arbeitserfahrung auf einer Palliativstation
- [ ] über ein Jahr Arbeitserfahrung in der SAPV
- [ ] über ein Jahr Arbeitserfahrung in der Intensivmedizin

Haben Sie Weiterbildung(en) oder Kurse in der Palliativmedizin / in Palliative Care abgeschlossen?

Bitte wählen Sie eine der folgenden Antworten:

- ja
- nein
- in Ausbildung

Ist Ihr aktuell überwiegender Tätigkeitsbereich universitär angebunden?

Bitte wählen Sie eine der folgenden Antworten:

- ja
- nein

Verfügen Sie über internationale Arbeitserfahrung?

Bitte wählen Sie eine der folgenden Antworten:

- ja
- nein

Über wie viel Berufserfahrung in der Anwendung sedierender Medikamente bei schwerkranken und sterbenden Patient*innen verfügen Sie?

Bitte wählen Sie eine der folgenden Antworten:

- unter 5 Jahre
- 5-9 Jahre
- 10-19 Jahre
- 20-29 Jahre
- 30 oder mehr Jahre

7. **Abschlusstext**

Umfrage abschließen

Liebe*r Teilnehmer*in,

dies ist die letzte Seite der Umfrage. Wenn Sie nun unten rechts auf „Absenden“ klicken, werden Ihre Antworten an uns übertragen und können von Ihnen nicht mehr bearbeitet werden.

Sollten Sie davor noch eine Ihrer Antworten überarbeiten wollen, nutzen Sie bitte entweder den „Zurück“-Button unten links oder die Navigationshilfe oben rechts in der Umfrage.

8. Allgemeines zum Layout

8.1 Prozentleiste

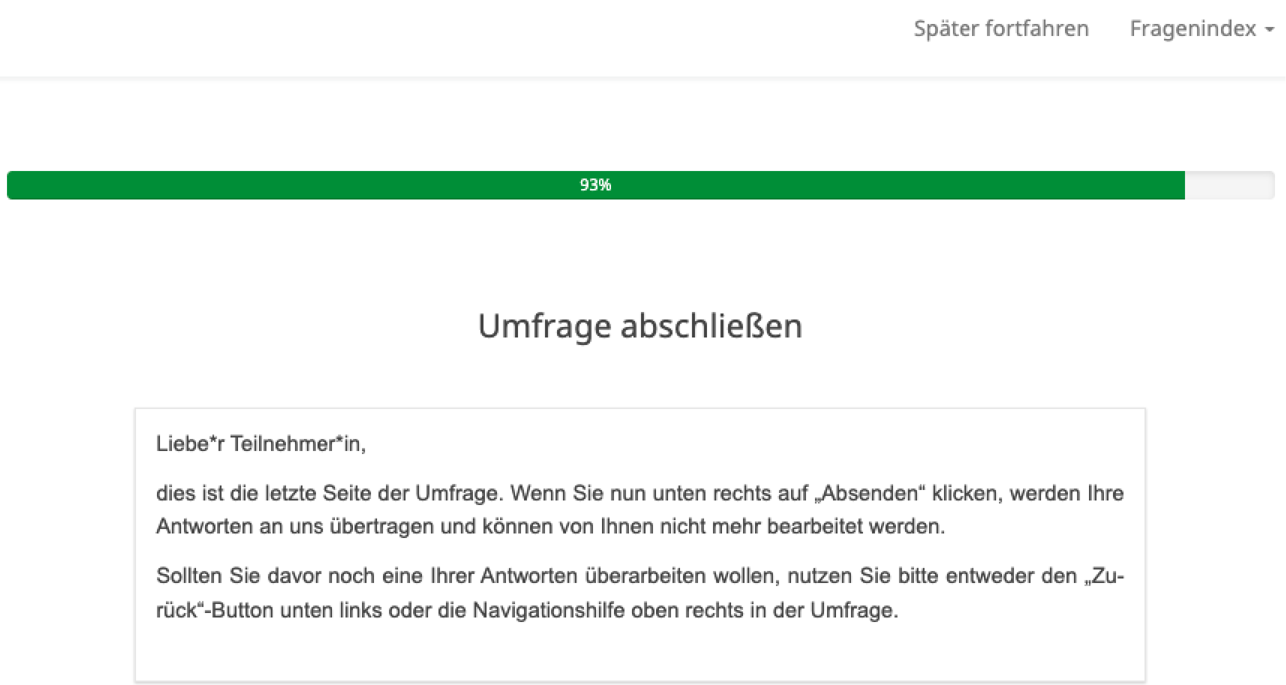

8.2 Navigationshilfe: Fragenindex

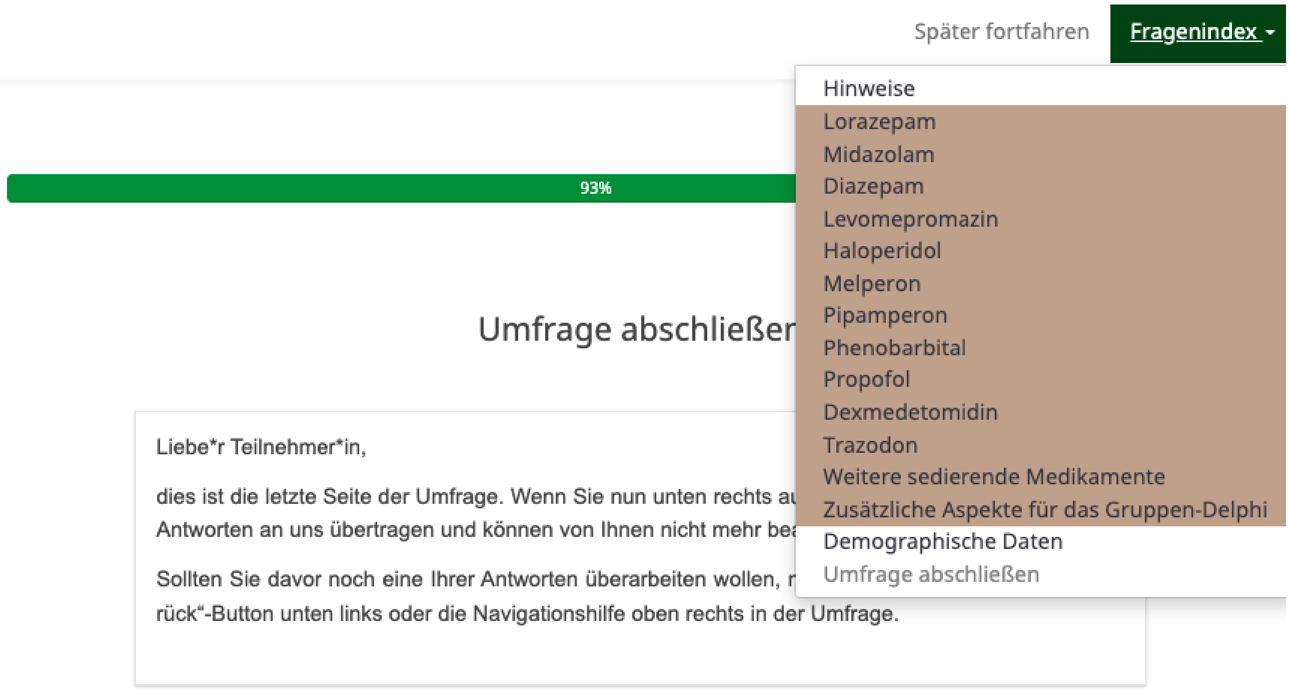

Supplement: Supplementary file 2 — Supplementary Material 2. [file 12904_2025_1781_MOESM2_ESM.pdf]
